# Substrate specificity of *Burkholderia pseudomallei* multidrug transporters is influenced by the hydrophilic patch in the substrate‐binding pocket

**DOI:** 10.1002/1873-3468.70248

**Published:** 2025-12-14

**Authors:** Ui Okada, Satoshi Murakami

**Affiliations:** ^1^ Department of Life Science and Technology Institute of Science Tokyo Yokohama Japan

**Keywords:** aminoglycosides, *Burkholderia pseudomallei*, multidrug resistance, RND transporter, substrate specificity

## Abstract

The Gram‐negative pathogen *Burkholderia pseudomallei* possesses multiple resistance‐nodulation‐division superfamily transporters that contribute to multidrug resistance, including BpeB and BpeF. Structural studies of BpeB and BpeF have identified a hydrophilic patch in their substrate‐binding pocket. To investigate the relationship between this hydrophilic patch and substrate specificity, mutant analyses were performed using an *Escherichia coli* recombinant expression system. Drug susceptibility tests of BpeB and BpeF mutants showed up to a 64‐fold increase in susceptibility compared with the wild type. Growth curve analyses revealed that BpeB mutants exhibited increased resistance to aminoglycosides, which are not transported by the wild type. These findings suggest that the hydrophilic patches in the substrate‐binding pockets of BpeB and BpeF are involved in the substrate specificity.

## Graphical abstract

Multidrug transporters BpeB and BpeF from the Gram‐negative pathogen *Burkholderia pseudomallei* have a hydrophilic patch in their substrate‐binding pocket. Drug susceptibility tests and growth curve analyses using an *Escherichia coli* recombinant expression system revealed that the hydrophilic patches of BpeB and BpeF are involved in the substrate specificity.

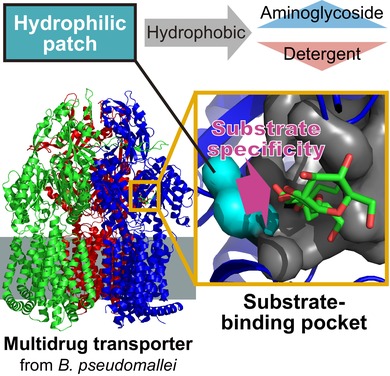

## Abbreviations

ACR, acriflavine

CHL, chloramphenicol

CLI, clindamycin

Cryo‐EM, cryo‐electron microscopy

DBP, distal binding pocket

DDM, n‐dodecyl‐β‐D‐maltoside

EB, ethidium bromide

ERY, erythromycin

GEN, gentamicin

KAN, kanamycin

LVX, levofloxacin

MD, molecular dynamics

MFP, membrane fusion protein

MIC, minimum inhibitory concentration

NOR, norfloxacin

OMC, outer membrane channel

PBP, proximal binding pocket

RND, resistance‐nodulation‐division

TET, tetracycline

TMP, trimethoprim

TOB, tobramycin

UDM, n‐undecyl‐β‐D‐maltoside

WT, wild type

*Burkholderia pseudomallei* is a Gram‐negative soil pathogen and the causative agent of melioidosis [1, 2, 3, 4, 5]. Melioidosis is a fatal infectious disease that has mainly been reported in tropical and subtropical countries; however, there are also many cases of infection among travelers to these regions, thus making it a global health issue. *B. pseudomallei* is intrinsically resistant to various antibiotics, including β‐lactams, macrolides, fluoroquinolones, and aminoglycosides, making melioidosis difficult to treat [2, 5]. Several factors contribute to the multidrug resistance of *B. pseudomallei*, including enzymatic inactivation of drugs and altered target sites, but one of the most crucial factors is the active efflux of drugs from the cells via transporters such as the resistance‐nodulation‐division (RND) superfamily transporters [2, 6, 7]. The RND superfamily transporter is largely driven by the proton motive force, which forms a tripartite complex that spans the inner membrane and outer membrane with the membrane fusion protein (MFP) and outer membrane channel (OMC) [8, 9, 10, 11, 12, 13]. The tripartite complex pumps out various substrates from the outer leaflet of the inner membrane and periplasm to the outside of the outer membrane. The *B. pseudomallei* prototype strain K96243 chromosomally encodes 10 RND superfamily transporters [14]. To date, three multidrug transporters of the RND superfamily, AmrB, BpeB, and BpeF, have been characterized [15]. The *amrB*, *bpeB*, and *bpeF* genes encoding RND‐type transporters form operons with *amrA*, *bpeA*, and *bpeE*, which encode MFPs, and *oprA*, *oprB*, and *oprC*, which encode OMCs, respectively, and subsequently their gene products from each operon assemble in the tripartite complexes. The AmrA–AmrB–OprA complex was identified to confer aminoglycoside and macrolide resistance [16, 17, 18, 19, 20]. The BpeA–BpeB–OprB complex exports chloramphenicol (CHL), fluoroquinolones, and macrolides [21, 22], and the BpeE–BpeF–OprC complex exports CHL, fluoroquinolones, and trimethoprim (TMP) [23, 24]. As detailed above, the three tripartite complexes of *B. pseudomallei* have some common substrates that they export, but the substrate specificity of these complexes is different. It is noteworthy that AmrA–AmrB–OprA can export aminoglycosides, but BpeA–BpeB–OprB and BpeE–BpeF–OprC cannot.

**Fig. 1:**
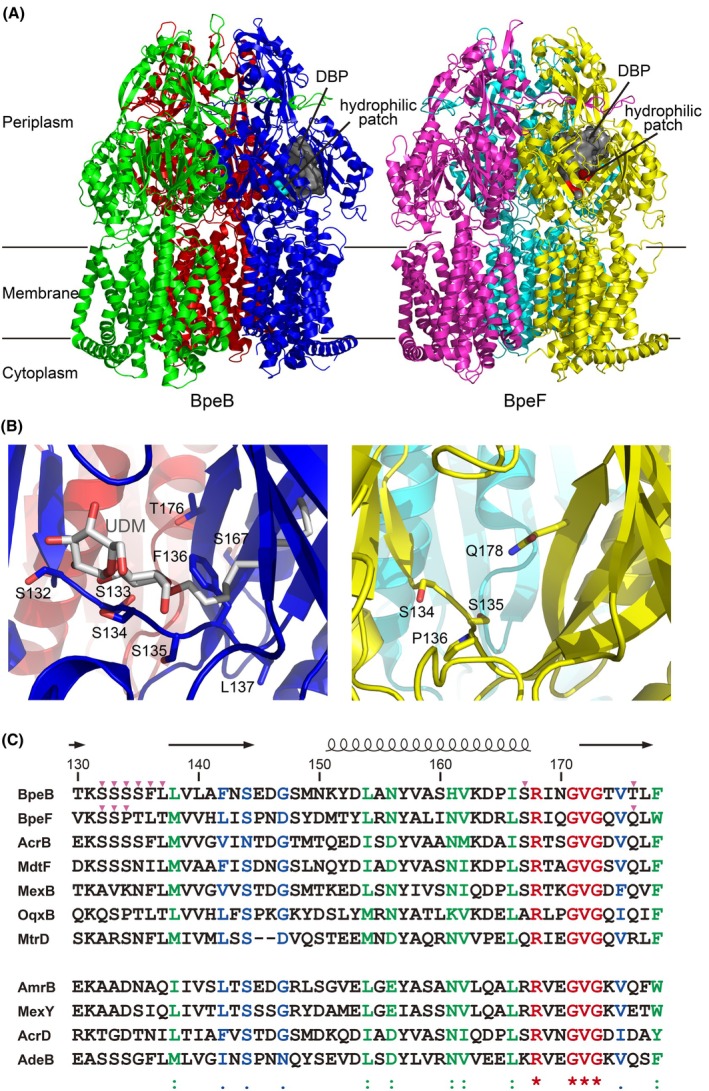
Structures of BpeB and BpeF. (A) Cartoon representations of the crystal structure of the BpeB (left) [PDB ID: 7WLS] and BpeF (right) [PDB ID: 7WLV] [25] trimers viewed parallel to the membrane plane. The three monomers of BpeB are colored green, blue, and red, respectively, and the three monomers of BpeF are colored magenta, yellow, and cyan, respectively. The DBP of the binding‐state monomer is shown as a gray surface. Pockets were detected using the Fpocket program [48]. The hydrophilic patch within the DBP is colored cyan for BpeB and red for BpeF. (B) Close‐up view of the DBP in the binding‐state monomer of BpeB (left) and BpeF (right). The bound UDM molecule and side chains of the residues forming the hydrophilic patch, which were mutated in this study, are shown in the stick representation. In the stick representation, the atoms are colored as follows: O, red; N, bright blue; C, gray (UDM), blue (BpeB), and yellow (BpeF). (C) The amino acid sequence alignment around the hydrophilic patch. The amino acid sequences of AmrB (UniProt ID: Q63U14), BpeB (Q63WS7), and BpeF (Q63NK6) from *B. pseudomallei*; AcrB (P31224), AcrD (P24177), and MdtF (P37637) from *E. coli*; MexB (P52002) and MexY (G3XCW2) from *Pseudomonas aeruginosa*; OqxB (A6TCQ5) from *Klebsiella pneumoniae*; MtrD (Q5F725) from *Neisseria gonorrhoeae*; and AdeB (A0A7U3Y749) from *A. baumannii* were aligned using ClustalW multiple sequence alignment [49]. Of these, the four in the bottom row (AmrB, MexY, AcrD, and AdeB) have been reported to be involved in aminoglycoside resistance [16, 37, 38, 39, 40]. The letters in red, green, and blue are identical, strongly similar, and weakly similar amino acids, respectively. The secondary structure elements of BpeB are shown on the top as a coil and arrows for the α‐helix and β‐strands, respectively. The magenta triangles indicate the residues that were mutated in this study.

Previously, we determined the crystal structures of the RND multidrug transporters BpeB and BpeF from *B. pseudomallei* [25]. Similar to other RND transporters, BpeB and BpeF each had two substrate‐binding pockets, the proximal binding pocket (PBP), also referred to as the access pocket, and the distal or deep binding pocket (DBP) in the periplasmic porter domain. The PBP is closer to the inner membrane and is located near the entrance of the substrate translocation pathway. The DBP is separated from the PBP by a switch loop consisting of 11 amino acids that undergo a conformational change during the transport process [26] and is located closer to the central funnel of homotrimeric BpeB or BpeF, which represents the exit of the substrate translocation pathway. In the DBP, multiple aromatic residues provide π–π stacking and hydrophobic interactions for substrate recognition. In addition, the DBP has a hydrophilic surface that is formed by hydrophilic residues (S132, S133, S134, S135, and T176 in BpeB; S134, S135, T137, T139, and Q178 in BpeF) (Fig. 1A,B) [25]. Furthermore, the S134 side chain that composes the hydrophilic patch forms a hydrogen bond with the hydrophilic head group of the n‐undecyl‐β‐D‐maltoside (UDM) in the UDM‐bound BpeB structure (Fig. 1B), and these residues of the hydrophilic patch are likely important for the recognition of the hydrophilic moiety of the amphiphilic substrates. In AmrB, some of these hydrophilic residues are substituted by hydrophobic residues (Fig. 1C), although AmrB exports hydrophilic aminoglycosides. This suggests that the hydrophilic patch may contribute to the substrate specificity. Compared with the studies on the role of hydrophobic residues in the DBP, those considering the hydrophilic residues have been insufficient. To investigate the importance of the hydrophilic patch in the DBP, we constructed its mutants and analyzed their drug susceptibility.

## Materials and methods

### Plasmid construction and transformation

The primers used for gene amplification and point mutation are summarized in Table S1. The *amrA* (NCBI Locus Tag, BPSL1804), *amrB* (NCBI Locus Tag, BPSL1803), and *oprA* (NCBI Locus Tag, BPSL1802) genes were amplified by PCR using synthesized expression vectors of each gene as templates, which were kindly provided by Dr. Thomas C. Terwilliger (Los Alamos National Laboratory). Depending on the primer sequence, the resulting PCR products contained restriction enzyme sites at the 5′‐ and 3′‐sides (*Eco*R I and *Nco* I for the *amrA* gene; *Nco* I and *Xba* I for the *amrB* gene; *Xba* I and *Hin*d III for the *oprA* gene). These genes were sequentially inserted into the pBAD24 vector [27] by digestion with the restriction enzymes, followed by ligation. The genes were ordered *amrA*–*amrB*–*oprA*, and the restriction enzyme sites *Nco* I and *Xba* I between them were designed with synonymous codon substitutions; thus, no extra sequence was added. The sequence of the linker between the *amrA* and *amrB* genes and the overlapping of the *amrB* and *oprA* genes were designed to match those of the genomic DNA.

Point mutations of AmrB, BpeB, and BpeF were introduced using an inverse PCR‐based site‐directed mutagenesis kit with KOD DNA polymerase (TOYOBO, Osaka, Japan) and the primers listed in Table S1. The pBAD24 vectors containing *amrA*–*amrB*–*oprA*, *bpeA*–*bpeB*–*oprB*, or *bpeE*–*bpeF*–*oprC* were used as PCR templates.

All constructed plasmids were verified by DNA sequencing, and each of the constructed plasmids was transformed into *E. coli* W3104*ΔacrABD* [28].

### Western blot analysis

*E. coli* W3104*ΔacrABD* harboring a plasmid encoding the wild type (WT) or mutants was cultivated at 37 °C with shaking at 120 rpm in Davis minimal medium [29] supplemented with 0.2% (w/v) glucose and 0.1% (w/v) casamino acid. Protein expression was induced for 3 h by the addition of 2% (w/v) L‐arabinose. The cells were harvested, resuspended in 50 mm Tris/HCl (pH 7.0) and 10% (v/v) glycerol, and disrupted by sonication. Cell debris was removed by centrifugation at 14 000 ***g*** for 10 min. The supernatant was collected and ultracentrifuged at 220 000 ***g*** for 30 min. The precipitates as the plasma membrane fraction were resuspended in 1% (w/v) sodium dodecyl sulfate, and the total protein concentration of the membrane fraction was determined using the bicinchoninic acid protein assay (ThermoFisher Scientific, Waltham, MA, USA).

Aliquots of the plasma membrane fractions were loaded and separated via sodium dodecyl sulfate polyacrylamide gel electrophoresis. After transfer to a polyvinylidene difluoride membrane (Amersham Hybond P, Cytiva, Uppsala, Sweden), the blots were blocked with 5% (w/v) skim milk in Tris‐buffered saline (10 mm Tris/HCl pH 7.5, 150 mm NaCl, and 0.05% (v/v) Tween 20). The proteins were detected using mouse monoclonal anti‐BpeB antibody (1 : 1000 dilution) or rabbit polyclonal anti‐BpeF antibody (1 : 4000 dilution) as the primary antibodies and horseradish peroxidase‐labeled goat anti‐mouse IgG (1 : 40 000 dilution, cat# 115‐035‐062, Jackson ImmunoResearch, West Grove, PA, USA) or anti‐rabbit IgG (1 : 20 000 dilution, cat# 111‐035‐003, Jackson ImmunoResearch) as the secondary antibodies, respectively. The bound antibodies were detected using Clarity Western ECL Substrate (Bio‐Rad, Hercules, CA, USA).

### Nile red efflux assay

Overnight cultures of *E. coli* W3104*ΔacrABD* harboring a plasmid encoding the WT or mutants were inoculated into 10 mL of Davis minimal medium supplemented with 0.2% (w/v) glucose and 0.1% (w/v) casamino acid and cultivated at 25 °C with shaking at 120 rpm. Protein expression was induced for 3 h by the addition of 2% (w/v) L‐arabinose at an OD_600_ of 0.4. The cells were harvested from 1 mL culture and resuspended in 1 mL of 20 mm potassium phosphate buffer (pH 7.0) containing 1 mm MgCl_2_ (PPB). Carbonyl cyanide *m*‐chlorophenylhydrazone and Nile red were added to final concentrations of 100 μm and 5 μm, respectively. After incubation at 37 °C for 5 min, cells were pelleted by centrifugation at 7000 ***g*** for 2 min and resuspended in 2 mL of PPB. Nile red fluorescence was measured in a fluorescence spectrophotometer (F‐7000; Hitachi High‐Tech, Tokyo, Japan) with excitation at 552 nm and emission at 636 nm. Nile red efflux was triggered by the addition of glucose to a final concentration of 0.2% (w/v).

### Drug susceptibility agar plate assay

Overnight cultures of *E. coli* W3104*ΔacrABD* harboring a plasmid encoding the WT or mutants were inoculated into 100 μL of 2× YT medium [1.6% (w/v) tryptone, 1% (w/v) yeast extract, and 0.5% (w/v) NaCl] containing 100 μg·mL^−1^ of ampicillin and cultivated at 37 °C with shaking at 600 rpm for 5 h. To induce protein expression, 2% (w/v) L‐arabinose was added, and the cultures were incubated for another 30 min with shaking. Cultures were inoculated onto YT agar plates [0.8% (w/v) tryptone, 0.5% (w/v) yeast extract, 0.5% (w/v) NaCl, 1.5% (w/v) agar, 100 μg·mL^−1^ ampicillin, and 2% (w/v) L‐arabinose] with sequential dilutions of erythromycin (ERY), clindamycin (CLI), tetracycline (TET), CHL, levofloxacin (LVX), norfloxacin (NOR), TMP, acriflavine (ACR), ethidium bromide (EB), n‐dodecyl‐β‐D‐maltoside (DDM), UDM, gentamicin (GEN), kanamycin (KAN), and tobramycin (TOB). The agar plates were incubated at 30 °C for 16 h. Minimum inhibitory concentrations (MICs) were determined visually as the lowest concentration at which no colonies were formed. The assays were repeated at least three times.

### Measurement of the cellular growth rate

Overnight cultures from single colonies of susceptible *E. coli* W3104*ΔacrABD*, harboring a plasmid encoding the WT or mutants summarized in Table 1, were grown in 2× YT medium containing 100 μg·mL^−1^ of ampicillin. Cultures were inoculated into Davis minimal medium supplemented with 0.2% (w/v) glucose and 0.1% (w/v) casamino acids and grown for approximately 16 h at 37 °C. The cells were then diluted to an OD_600_ of 0.2 in fresh medium in the wells of a 96‐well plate to which serially diluted concentrations of GEN or TOB were added. L‐arabinose was added to a final concentration of 2% (w/v) to induce protein expression. The microplate was incubated at 25 °C with shaking at 600 rpm and measured at OD_600_ in a VersaMax microplate reader (Molecular Devices, San Jose, CA, USA). The assay was repeated at least three times.

**Table 1:** Bacterial strain and plasmids used in this study.

Plasmids	Characteristics	Source or reference
pBAD24	Expression vector, P_BAD_ promoter, Ampicillin resistance	[27]
pAB_WT	pBAD24‐based expression vector containing *amrA*–*amrB*–*oprA* (WT)	This study
pAB_DN	pAB_WT with D406N and D407N mutations in AmrB. (transport‐inactive mutant, negative control)	This study
pBB_WT	pBAD24‐based expression vector containing *bpeA*–*bpeB*–*oprB* (WT)	[25]
pBB_DN	pBB_WT with D407N and D408N mutations in BpeB. (transport‐inactive mutant, negative control)	[25]
pB_S132A	pBB_WT with S132A mutation in BpeB	This study
pB_S132A + S133A	pBB_WT with S132A and S133A mutations in BpeB	This study
pB_S132A + S133A +S134D	pBB_WT with S132A, S133A, and S134D mutations in BpeB	This study
pB_T176V	pBB_WT with T176V mutation in BpeB	This study
pB_S132A + S133A +S134D + T176E	pBB_WT with S132A, S133A, S134D, and T176E mutations in BpeB	This study
pB_S132A‐L137Q	pBB_WT with S132A, S133A, S134D, S135N, F136A, and L137Q mutations in BpeB	This study
pB_S167R	pBB_WT with S167R mutation in BpeB	This study
pBF_WT	pBAD24‐based expression vector containing *bpeE*–*bpeF*–*oprC* (WT)	[25]
pBF_DN	pBF_WT with D410N and D411N mutations in BpeF. (transport‐inactive mutant, negative control)	[25]
pF_S134A	pBF_WT with S134A mutation in BpeF	This study
pF_S134A + S135A	pBF_WT with S134A and S135A mutations in BpeF	This study
pF_S134A + S135A + P136D	pBF_WT with S134A, S135A, and P136D mutations in BpeF	This study
pF_Q178V	pBF_WT with Q178V mutation in BpeF	This study
pF_S134A + S135A + Q178V	pBF_WT with S134A, S135A, and Q178V mutations in BpeF	This study

Strain	Characteristics	Reference
*E. coli* W3104 *ΔacrABD*	Drug‐susceptible strain	[28]

## Results

### Substrate specificity of AmrB

For a positive control of aminoglycoside efflux activity, an *Escherichia coli* expression system of AmrA–AmrB–OprA from *B. pseudomallei* was constructed. *E. coli* expressing AmrA–AmrB–OprA exhibited a 2‐ to 64‐fold increased resistance to ERY, CLI, TET, LVX, TMP, ACR, EB, DDM, and UDM compared with the negative control (Table 2, Fig. S1). Previous reports have shown that AmrB is not involved in TET or fluoroquinolone resistance [16], but in our experiments, a twofold increase in resistance to TET and LVX was observed. In a previous study, the susceptible strains of *B. pseudomallei* were used, but in this study, recombinant *E. coli* strains were used, which may have influenced this difference. TMP, DDM, and UDM have not been previously reported as substrates and are therefore newly identified as AmrB substrates. The rest of the substrates are in good agreement with previous MIC measurement studies using the gene‐disrupted strain of *B. pseudomallei* [16, 18]. However, the expression of AmrA–AmrB–OprA did not influence the susceptibilities of CHL and NOR, which is consistent with a previous study [16].

**Table 2:** Measurement of the drug resistance of *E. coli* W3104*ΔacrABD* expressing *B. pseudomallei* AmrA–AmrB–OprA, BpeA–BpeB–OprB, and BpeE–BpeF–OprC and their AmrB, BpeB, and BpeF mutants. The resistance levels are shown as the minimum inhibitory concentration (MIC) in μg·mL^−1^ determined using the agar dilution method. Values are based on data shown in Figs S1, S4, and S5. To induce protein expression, 2% (w/v) L‐arabinose was added to the agar plates. Details of the plasmids contained are summarized in Table 1. ACR, acriflavine; CHL, chloramphenicol; CLI, clindamycin; DDM, n‐dodecyl‐β‐D‐maltoside; EB, ethidium bromide; ERY, erythromycin; GEN, gentamicin; KAN, kanamycin; LVX, levofloxacin; NOR, norfloxacin; TET, tetracycline; TMP, trimethoprim; TOB, tobramycin. UDM, n‐undecyl‐β‐D‐maltoside. Positive results (orange shadings) indicate increased resistance to the drug compared with the negative control (empty vector). The assay was repeated at least three times to ensure that all results were consistent.

Containing plasmid	MIC [μg·mL^−1^]													
	ERY	CLI	TET	CHL	LVX	NOR	TMP	ACR	EB	DDM	UDM	GEN	KAN	TOB
pBAD24	6.25	3.13	0.39	0.78	0.006	0.025	0.10	6.25	12.5	62.5	125	6.25	6.25	6.25
pAB_WT	100	50.0	0.78	0.78	0.012	0.025	0.20	25.0	100	> 4000	> 4000	3.13	3.13	3.13
pAB_DN	6.25	3.13	0.39	0.78	0.006	0.025	0.10	6.25	12.5	62.5	125	3.13	3.13	3.13
pBB_WT	25.0	25.0	0.78	1.56	0.012	0.025	0.20	12.5	25.0	> 4000	> 4000	6.25	6.25	6.25
pBB_DN	6.25	3.13	0.39	0.78	0.006	0.025	0.10	6.25	12.5	62.5	125	6.25	6.25	6.25
pB_S132A	25.0	12.5	0.78	1.56	0.012	0.025	0.20	12.5	25.0	> 4000	> 4000	6.25	6.25	6.25
pB_S132A + S133A	25.0	12.5	0.78	1.56	0.012	0.025	0.20	12.5	25.0	> 4000	> 4000	6.25	6.25	6.25
pB_S132A + S133A + S134D	25.0	25.0	0.78	1.56	0.012	0.025	0.20	12.5	25.0	> 4000	> 4000	6.25	6.25	6.25
pB_T176V	25.0	12.5	0.78	1.56	0.012	0.025	0.20	12.5	25.0	> 4000	> 4000	6.25	6.25	6.25
pB_S132A + S133A + S134D + T176E	12.5	12.5	0.78	1.56	0.012	0.025	0.20	12.5	25.0	> 4000	1000	6.25	6.25	6.25
pB_S132A‐L137Q	12.5	25.0	0.78	1.56	0.012	0.025	0.20	12.5	25.0	> 4000	> 4000	6.25	6.25	6.25
pB_S167R	12.5	6.25	0.78	1.56	0.006	0.025	0.20	12.5	12.5	125	250	6.25	6.25	6.25
pBF_WT	6.25	12.5	0.39	6.25	0.012	0.025	0.20	12.5	25.0	> 4000	500	6.25	6.25	6.25
pBF_DN	6.25	3.13	0.39	0.78	0.006	0.025	0.10	6.25	6.25	31.3	62.5	3.13	3.13	3.13
pF_S134A	6.25	6.25	0.39	6.25	0.012	0.025	0.20	12.5	25.0	> 4000	500	6.25	6.25	6.25
pF_S134A + S135A	6.25	6.25	0.39	3.13	0.012	0.025	0.20	12.5	25.0	> 4000	250	6.25	6.25	6.25
pF_S134A + S135A + P136D	6.25	3.13	0.39	1.56	0.006	0.025	0.10	12.5	12.5	62.5	125	6.25	3.13	3.13
pF_Q178V	6.25	6.25	0.39	6.25	0.006	0.025	0.20	12.5	25.0	> 4000	250	6.25	3.13	3.13
pF_S134A + S135A + Q178V	6.25	6.25	0.39	3.13	0.012	0.025	0.20	12.5	25.0	> 4000	250	6.25	6.25	6.25

A major difference between the reported results [16, 17, 18, 19, 20] and our MIC measurement results on plate cultures using recombinant *E. coli* is that the expression of AmrA–AmrB–OprA did not affect resistance to aminoglycosides such as GEN, KAN, and TOB.

### Mutations around the hydrophilic patch

To investigate the importance of the hydrophilic patch in the DBP, a series of mutations in BpeB and BpeF were constructed in which the hydrophilic residues were substituted with hydrophobic residues in the same manner as AmrB (Table 1, Fig. 1C). In addition, considering the possibility that residues around the hydrophilic patch may also affect the substrate specificity, we constructed mutants in which these amino acids were substituted with the same amino acids as in AmrB. All mutants were expressed as well as the BpeB or BpeF WTs (Fig. S2). Furthermore, the Nile red efflux assay showed that all BpeB and BpeF mutants retained efflux activity toward Nile red, indicating that the introduced mutations did not abolish the efflux function (Fig. S3). While some mutants showed modest differences in dye efflux activity, all mutants retained measurable transport activity.

First, *E. coli* harboring pB_S132A, pB_S132A + S133A, and pB_T176V, in which the hydrophilic residues of BpeB were substituted with hydrophobic amino acids, exhibited a twofold reduced CLI resistance compared with the WT (Table 2). Furthermore, mutants around the hydrophilic patch revealed reduced resistance to ERY and UDM as well as to CLI. In particular, the S167R mutant showed a 4‐ to 16‐fold reduction in resistance to CLI and detergents such as DDM and UDM. Among the mutants tested in this study, the resistance to TET, CHL, TMP, and ACR was equivalent to that of the WT. Furthermore, all of the mutants used in this study were equally sensitive to NOR and aminoglycosides, such as GEN, KAN, and TOB, which have no efflux activity in the WT.

Second, in the case of BpeF, the mutants of the hydrophobic substitutions applied to pF_S134A, pF_S134A + S135A, pF_Q178V, and pF_S134A + S135A + Q178V showed a twofold reduced resistance to CLI, CHL, and UDM compared with the WT (Table 2). The pF_S134A + S135A + P136D mutant showed a 2‐ to 64‐fold reduced resistance to CLI, CHL, LVX, TMP, EB, DDM, and UDM. All mutants retained resistance to ACR comparable to that of the WT and, similar to the WT, they were not resistant to ERY, TET, NOR, and aminoglycosides.

### Aminoglycoside susceptibility

The MIC measurements were determined by colony formation on agar plates and revealed no difference in aminoglycoside susceptibility among all the constructed mutants (Table 2, Figs S4 and S5). Moreover, in our system, no aminoglycoside resistance was observed in the WT of AmrB, which was used as a positive control (Table 2, Fig. S1). Therefore, we compared the growth curves to detect more subtle differences.

**Fig. 2:**
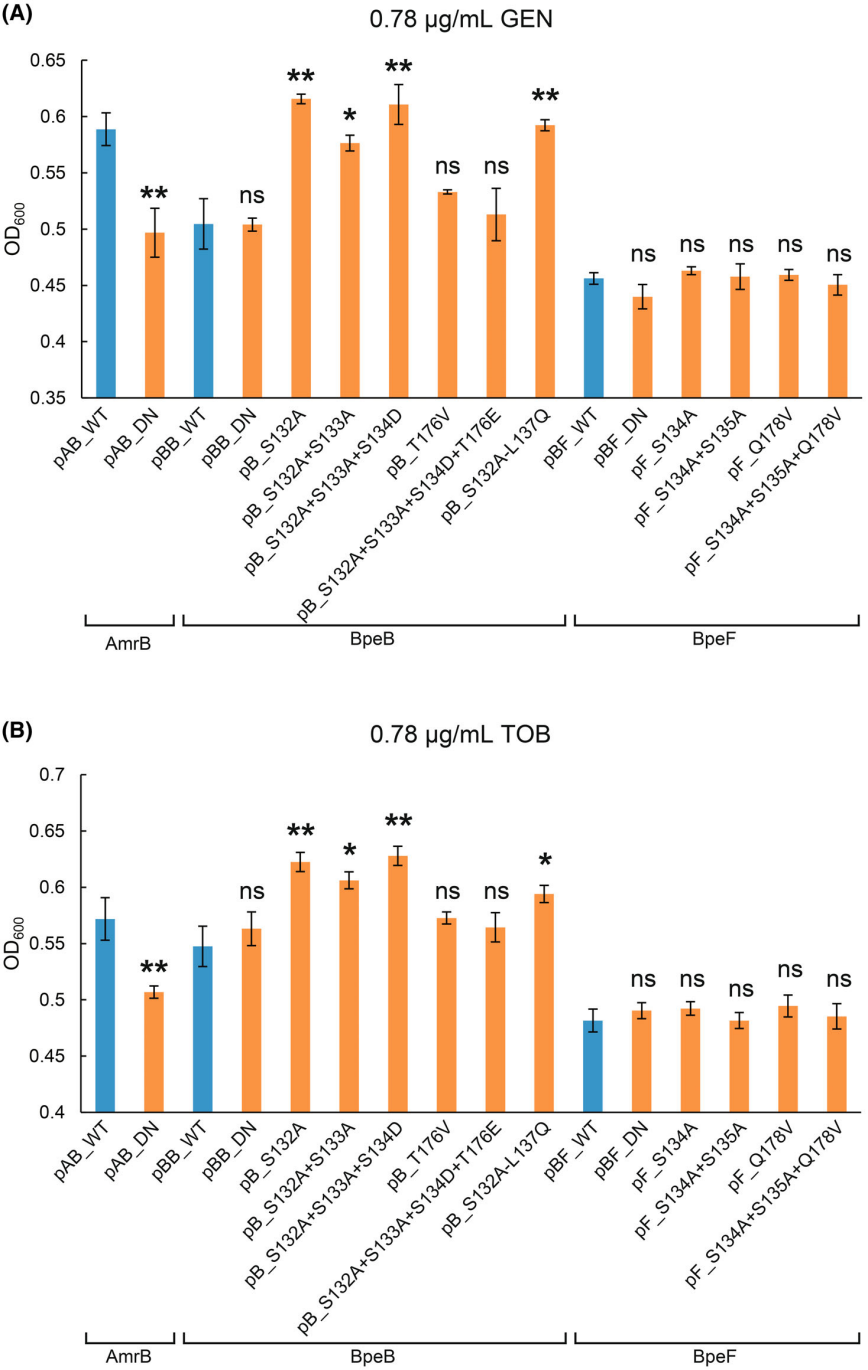
Growth comparison in the presence of aminoglycosides. The growth of *E. coli* W3104*ΔacrABD* harboring a plasmid expressing the WT (sky blue) or mutants (orange) in the presence of 0.78 μg·mL^−1^ of GEN (A) and TOB (B) is shown by the OD_600_ 4 h after induction. The details of the plasmids contained in *E. coli* are given in Table 1. The growth curves for each strain from which these data were derived are shown in Figs S6 and S7. The data are presented as the average of three independent experiments. Error bars indicate the standard deviation. *P*‐values were calculated using the two‐way Student's *t*‐test comparing the WT and mutant data sets (*P < 0.05; **P < 0.01; ns, not significant).

When comparing the growth curves of the WT and the transport‐inactive mutant of AmrB that are denoted as pAB_WT and pAB_DN, respectively, there was a clear difference in growth in the presence of aminoglycosides such as GEN and TOB (Fig. 2, Figs S6A, and S7A). However, there was no difference in growth between the WTs and the transport‐inactive mutants of BpeB and BpeF (Fig. 2, Figs S6B,I and S7B,I), indicating that they were not excreting aminoglycosides. These results are in good agreement with previous reports [15], and these findings confirm that this experimental system worked well.

Using this experimental system, the growth curves of the BpeB and BpeF mutants were compared. Remarkably, although the WT of BpeB did not export aminoglycosides, the BpeB mutants in the hydrophilic patch, such as pB_S132A, pB_S132A + S133A, pB_S132A + S133A + S134D, and pB_S132A‐L137Q, grew as well as the AmrB WT in the presence of GEN or TOB (Fig. 2, Figs S6C–E,H and S7C–E,H). The results indicate that these mutations enable BpeB to export aminoglycosides. On the other hand, the growth curves of *E. coli* harboring pB_T176V and pB_S132A + S133A + S134D + T176E were comparable to those of the WT, and no enhanced growth was observed (Fig. 2, Figs S6F,G and S7F,G). These results were similar for the two aminoglycosides. In contrast, for BpeF, the mutations around the hydrophilic patch constructed in this study did not influence the growth curves compared with the WT for both GEN and TOB (Fig. 2, Figs S6J–M, and S7J–M).

## Discussion

In this study, we used recombinant expression systems in *E. coli* to measure the MICs and growth curves. Because *E. coli* and *B. pseudomallei* have different properties, such as drug permeability and other cooperating drug resistance mechanisms, it is unclear whether these mutations change the substrate specificity of *B. pseudomallei*. However, all of the mutants created in this study were expressed at the same levels as the WT in recombinant *E. coli*, allowing us to fairly evaluate the effects of the mutations and discuss the molecular mechanisms of substrate specificity for these transporters.

The results of the MIC measurements on the agar plates revealed that mutations around the hydrophilic patch of BpeB and BpeF influenced the substrate specificity. Multiple mutations of the hydrophilic amino acids in the hydrophilic patch, such as the pF_S134A + S135A and pF_S134A + S135A + Q178V mutants, showed a twofold reduced resistance to CHL and UDM than a single mutation, such as the pF_S134A and pF_Q178V mutants (Table 2). These results suggest that the degree of hydrophilicity of the DBP may affect the substrate selectivity. On the other hand, single and multiple mutations have an equal effect on CLI resistance. These results suggest that hydrophobic substitutions are differently affected depending on the substrate. In resistance to ERY, CLI, and UDM, a 2‐ to 4‐fold difference was observed between pB_S132A + S133A + S134D and pB_S132A + S133A + S134D + T176E in BpeB (Table 2), and the difference between the two mutants was the T176E mutation. These results indicate that T176, which constitutes the hydrophilic patch, is important for the recognition of these substrates. The crystal structure of the ERY‐bound AcrB revealed that S134 and S135 of AcrB formed hydrogen bonds with ERY [30]. These residues are in the substrate‐binding pocket of AcrB and form a hydrophilic patch. It has also been reported that the growth of the S134A and S135A double mutant of AcrB was inhibited in the presence of ERY compared to the WT [30], which supports our results. These results suggest that the amino acids in the hydrophilic patch of BpeB and BpeF play a crucial role in substrate selectivity.

Next, we discuss the mutants that exhibited a notable change in drug susceptibility. The S167R mutant in BpeB showed a 16‐ to 32‐fold increased susceptibility to detergents (Table 2). In contrast, even with this mutation, there were drugs such as TET, CHL, TMP, and ACR that showed resistance to the same degree as that of the WT, indicating that the structure of the mutant had not been grossly disrupted. The finding that this mutant also retained Nile red efflux activity further corroborates that its overall structure and transport function were preserved (Fig. S3). S167 in BpeB was located near the exit channel of the substrate translocation pathway leading from the DBP to the central funnel, which has been proposed for RND transporters such as AcrB from *E. coli* [9, 10, 31, 32]. The bulky residue near the exit gate may interfere with the efflux of larger substrates such as ERY, DDM, and UDM, whereas smaller substrates such as ACR, TMP, and CHL were not affected and revealed resistance comparable to that of the WT. These results provide reasonable support for the elucidation of the substrate translocation pathway obtained through the structural analysis of the RND superfamily transporters that include BpeB.

Among the BpeF mutants, the pF_S134A + S135A + P136D mutant showed increased susceptibility to many substrates, including CLI, CHL, LVX, TMP, EB, DDM, and UDM, compared with the pF_S134A + S135A mutant. In particular, the susceptibility to DDM increased by more than 64‐fold. The difference between the two mutants is the P136D mutation, which is a mutation to proline that greatly impacts the main chain structure of the protein, and it is thought that the structure around the mutated site changed compared to that of the WT, thus greatly affecting drug sensitivity. On the other hand, compared to the pBF_DN mutant, a transport‐inactive mutant of BpeF, the pF_S134A + S135A + P136D mutant exhibited a twofold increased resistance to several drugs such as CHL, ACR, EB, DDM, and UDM. This indicates that the overall structure of the mutant is well maintained enough to sustain the transport activity, although the environment and characteristics within the DBP were changed. These results also suggest that the hydrophilic patch and its surrounding residues considerably affect the substrate specificity.

The structure of GEN‐bound AcrD was determined by cryo‐electron microscopy (cryo‐EM), and the translocation pathway of GEN was predicted using molecular dynamics (MD) simulation based on the binding site of GEN [33]. The pathway is via the DBP, and our mutagenesis results support this. Moreover, growth inhibition has been reported for K131 and D174 mutations of AcrD in the presence of GEN compared with the WT. Furthermore, the cryo‐EM structure of MexY and MD simulations revealed that charged residues, such as E129, D133, and K175, are crucially important for substrate recognition [34]. These critical residues in AcrD and MexY are located near the sites that correspond to the hydrophilic patch of BpeB or BpeF. Furthermore, for MdtF, which also possesses a hydrophilic patch similar to those of BpeB and BpeF, MD simulations based on its cryo‐EM structure have revealed that residues S134 and Q176, which constitute the hydrophilic patch, are involved in the recognition of rhodamine 6G and linezolid [35]. These results support our mutagenesis studies, which show that the residues around the hydrophilic patch affect substrate selectivity.

Unexpectedly, although AdeB transports aminoglycosides, it also harbors a hydrophilic patch. Structural studies of AdeB, including those in ethidium‐bound states, revealed that the distal loop (residues 131–139), which constitutes part of the hydrophilic patch, undergoes significant conformational changes during the substrate transport cycle [36]. Moreover, MD simulations have indicated that this loop plays a critical role in substrate translocation. Taken together with reported evidence that the hydrophilic patch and its surrounding residues are involved in the substrate transport mechanism, our experimental finding that mutations in this region affect substrate specificity appears highly reasonable.

Next, we discuss the results from the comparison of the BpeB mutant growth curves, observing the aminoglycoside efflux activity. Although both mutants were single amino acid residue substitutions in the hydrophilic patch of BpeB, the S132A mutant enabled the efflux of aminoglycosides, whereas the T176V mutant did not (Fig. 2). Even if the degree of hydrophilicity was reduced by a hydrophobic substitution, it is natural that the effect will differ depending on the substitution location within the DBP. Among the mutants measured in this study, pB_T176V and pB_S132A + S133A + S134D + T176E did not export aminoglycosides (Fig. 2), and they have the T176 mutation in common. As mentioned above, this residue also played a considerable role in the recognition of ERY, CLI, and UDM, as revealed by the MIC measurements on the agar plates. These results suggest that T176 is highly likely to play a key role in aminoglycoside recognition in the pB_S132A, pB_S132A + S133A, pB_S132A + S133A + S134D, and pB_S132A‐L137Q mutants constructed in this study. On the other hand, in the RND transporters AmrB, MexY from *Pseudomonas aeruginosa*, AcrD from *E. coli*, and AdeB from *Acinetobacter baumannii*, which export aminoglycosides as the WT [16, 37, 38, 39, 40], the corresponding amino acids were glutamine, glutamic acid, or aspartic acid (Fig. 1C). Note that the difference between pB_S132A + S133A + S134D, which exports aminoglycosides, and pB_S132A + S133A + S134D + T176E, which does not, is the T176E mutation. If the single amino acid T176 is important for the recognition of aminoglycosides, substitution with glutamic acid, as in MexY, should allow aminoglycosides to be exported; however, it did not. These results suggest that the substrate recognition of BpeB and BpeF depends not only on the hydrophilic patch‐specific residues but also on the affinity of the entire substrate‐binding pocket. Previous studies on RND transporters, such as AcrB, have revealed that many residues in the PBP and DBP are involved in the recognition of their respective substrates [25, 30, 31, 41, 42, 43], and these findings support our results.

Even though both BpeB and BpeF mutants had been introduced in the hydrophilic patch and its surrounding area, we were able to confirm the transport of aminoglycosides in the BpeB mutants, but not in the BpeF mutants (Fig. 2). Considering these results, it is natural to surmise that the BpeB mutants were able to transport aminoglycosides because the combination of the mutated section and the original amino acids of the DBP in the substrate translocation pathway of BpeB creates an environment that is suitable for the recognition of aminoglycosides. In contrast, for BpeF, the combination of the mutated and original amino acids was not suitable for aminoglycoside transport. These results indicate that the substrate specificity of BpeB and BpeF is determined not only by specific residues that correspond to the hydrophilic patch but also by other regions within the substrate‐binding pocket. To examine the relationship between the hydrophilic patch and substrate specificity in other RND transporters, the characteristics of DBPs from structurally known RND transporters were summarized (Table S2). Although the presence or absence of a hydrophilic patch clearly distinguishes these transporters, no significant differences were observed in the DBP volume or overall hydrophobicity. Similarly, when transporters were classified based on their ability to transport aminoglycosides, no clear differences were observed in either DBP volume or hydrophobicity. These observations indicate that substrate recognition occurs through multiple regions within the substrate translocation pathway rather than a specific local feature such as a hydrophilic patch. This is consistent with the findings from the previous structural analyses discussed above and the MD simulations of RND transporters [44, 45, 46]. Such a mechanism is likely to represent a general feature of RND transporters, not limited to BpeB and BpeF analyzed in this study.

Although it may seem counterintuitive, the DBPs of AmrB and MexY, which export hydrophilic substrates, such as aminoglycosides, are enriched in hydrophobic amino acids, as previously noted [47]. Our results revealed that the mutation of the BpeB hydrophilic patch with hydrophobic amino acids enabled aminoglycoside transport. This suggests that substrate recognition by RND transporters is governed largely by the overall architecture and environment of the substrate‐binding pocket, rather than by a few specific residues. This loose substrate recognition by RND transporters is called “Goldilocks” affinity, which means that the interactions are not too weak and not too strong, because too strong interactions may reduce the substrate export efficiency [47]. It seems likely that this moderate interaction allows the RND superfamily transporters to recognize substrates at several sites in the substrate translocation pathway, thereby enabling them to transport diverse substrate types.

In conclusion, the hydrophilic patch and its surrounding amino acids in the DBP play a substantial role in the substrate specificity of BpeB and BpeF. These were via MIC measurements using agar plates and in the comparison of the growth curves of recombinant *E. coli*. Furthermore, our mutational analysis revealed that the specific key residues in the hydrophilic patch of BpeB and BpeF, as well as the overall environment and characteristics of the substrate‐binding pocket, determine the substrate specificity. This indicates that the substrate specificity of these transporters is affected by the entire substrate translocation pathway, where they transport substrates that they are able to transport and not those that they cannot. This result may be consistent with the inference that these transporters are not originally designed for drug efflux, but rather that bacteria have exploited these existing transporters to survive in the presence of the various newly developed drugs. Using our results as a steppingstone, further research may be able to derive valuable information for the design of specific inhibitors of RND transporters of *B. pseudomallei*, which may be beneficial for treating melioidosis.

## Author contributions

UO contributed to the conceptualization, funding acquisition, investigation, methodology, visualization, writing – original draft, and writing – review and editing. SM contributed to the conceptualization, funding acquisition, supervision, and writing – review and editing.

## Supporting information


**Fig. S1.** Measurement of the drug resistance of *Escherichia coli* expressing *Burkholderia pseudomallei* AmrA–AmrB–OprA and their AmrB mutant.
**Fig. S2.** Western blot analysis of the BpeB and BpeF mutants.
**Fig. S3.** Nile red efflux assay of the BpeB and BpeF mutants.
**Fig. S4.** Measurement of the drug resistance of *E. coli* expressing *B. pseudomallei* BpeA–BpeB–OprB and their BpeB mutants.
**Fig. S5.** Measurement of the drug resistance of *E. coli* expressing *B. pseudomallei* BpeE–BpeF–OprC and their BpeF mutants.
**Fig. S6.** Comparison of the bacterial growth curves in the presence of GEN.
**Fig. S7.** Comparison of the bacterial growth curves in the presence of TOB.
**Table S1.** Cloning primers.
**Table S2.** Structural characteristics of the DBPs of RND transporters.

## Data Availability

The data that support the findings of this study are available within the article and in the Supporting Information. In addition, the drug susceptibility agar plate assay data have been deposited in the Dryad Digital Repository (DOI: 10.5061/dryad.bnzs7h4qz).
