# Supplementary material for: Substrate specificity of Burkholderia pseudomallei multidrug transporters is influenced by the hydrophilic patch in the substrate‐binding pocket
Source: FEBS Lett. 2025 Dec 14;600(4):537–49. doi: 10.1002/1873-3468.70248 (PMC12926853; doi:10.1002/1873-3468.70248)
Supplement: Supplementary file 1 — Fig. S1. Measurement of the drug resistance of Escherichia coli expressing Burkholderia pseudomallei AmrA–AmrB–OprA and their AmrB mutant. Fig. S2. Western blot analysis of the BpeB and BpeF mutants. Fig. S3. Nile red efflux assay of the BpeB and BpeF mutants. Fig. S4. Measurement of the drug resistance of E. coli expressing B. pseudomallei BpeA–BpeB–OprB and their BpeB mutants. Fig. S5. Measurement of the drug resistance of E. coli expressing B. pseudomallei BpeE–BpeF–OprC and their BpeF mutants. Fig. S6. Comparison of the bacterial growth curves in the presence of GEN. Fig. S7. Comparison of the bacterial growth curves in the presence of TOB. Table S1. Cloning primers. Table S2. Structural characteristics of the DBPs of RND transporters. [file FEB2-600-537-s001.docx]

**Supplemental material**

**Substrate specificity of *Burkholderia pseudomallei* multidrug transporters is influenced by the hydrophilic patch in the substrate-binding pocket**

Ui Okada* and Satoshi Murakami*

Department of Life Science and Technology, Institute of Science Tokyo, Nagatsuta, Midori-ku, Yokohama 226-8501, Japan

*Corresponding author’s email address:

Ui Okada, [uiokada@life.isct.ac.jp](mailto:uiokada@life.isct.ac.jp); Satoshi Murakami, [murakami@life.isct.ac.jp](mailto:murakami@life.isct.ac.jp)


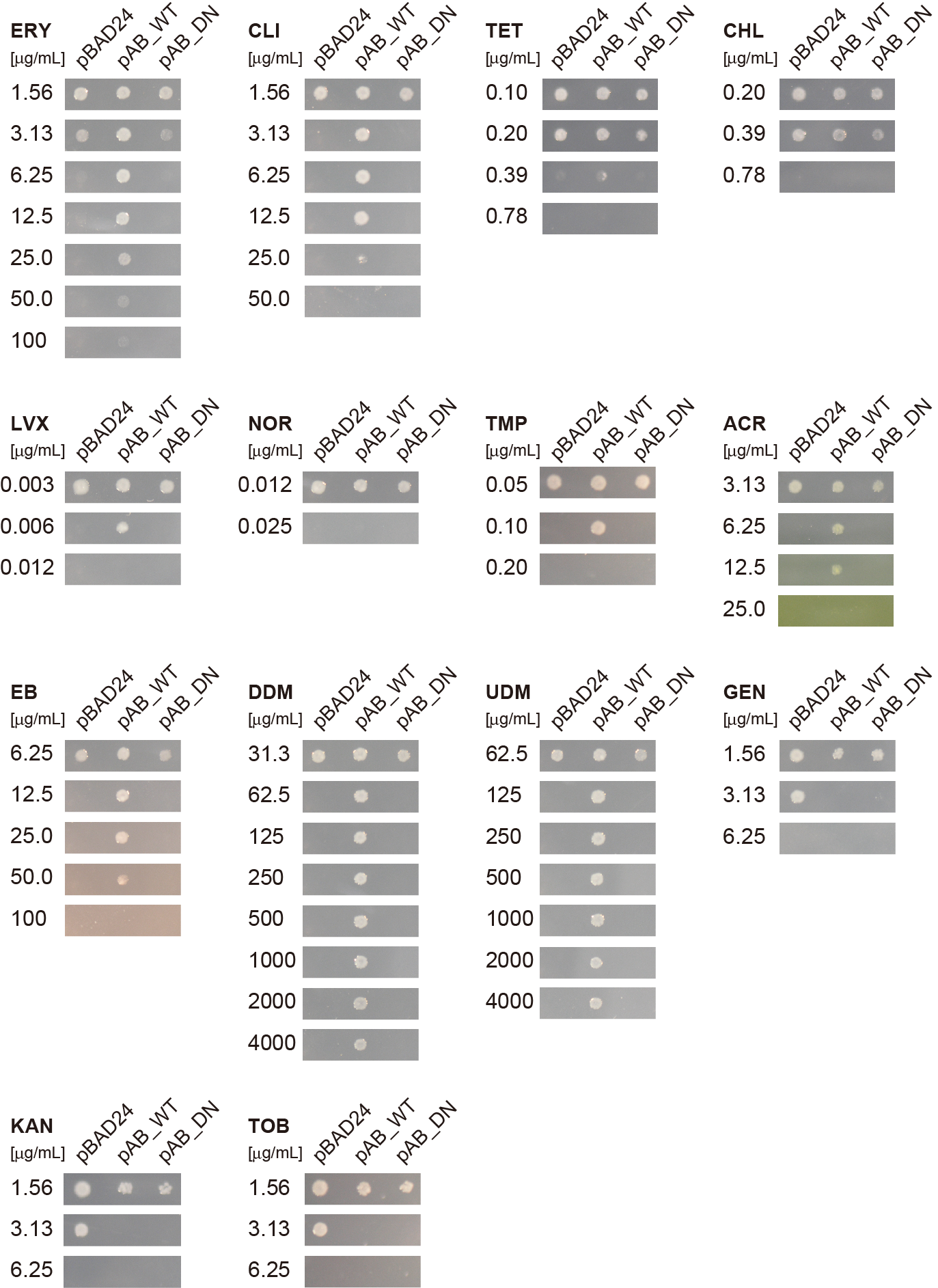


**Fig. S1 |** **Measurement of the drug resistance of *Escherichia coli* expressing *Burkholderia pseudomallei* AmrA–AmrB–OprA and their AmrB mutant**

For the minimum inhibitory concentration (MIC) measurements, cells were grown on agar plates in the presence of dilutions of the compounds. These concentrations are shown on the left-hand side of each panel. To induce protein expression, 2% (w/v) L-arabinose was added to the agar plates. The names of the plasmids contained in *E. coli* are written above each panel, and the details are provided in Table 1. ERY, erythromycin; CLI, clindamycin; TET, tetracycline; CHL, chloramphenicol; LVX, levofloxacin; NOR, norfloxacin; TMP, trimethoprim; ACR, acriflavine; EB, ethidium bromide; DDM, n-dodecyl-β-D-maltoside; UDM, n-undecyl-β-D-maltoside; GEN, gentamicin; KAN, kanamycin; TOB, tobramycin. Data in Table 2 are based on the colony formations shown in this figure.


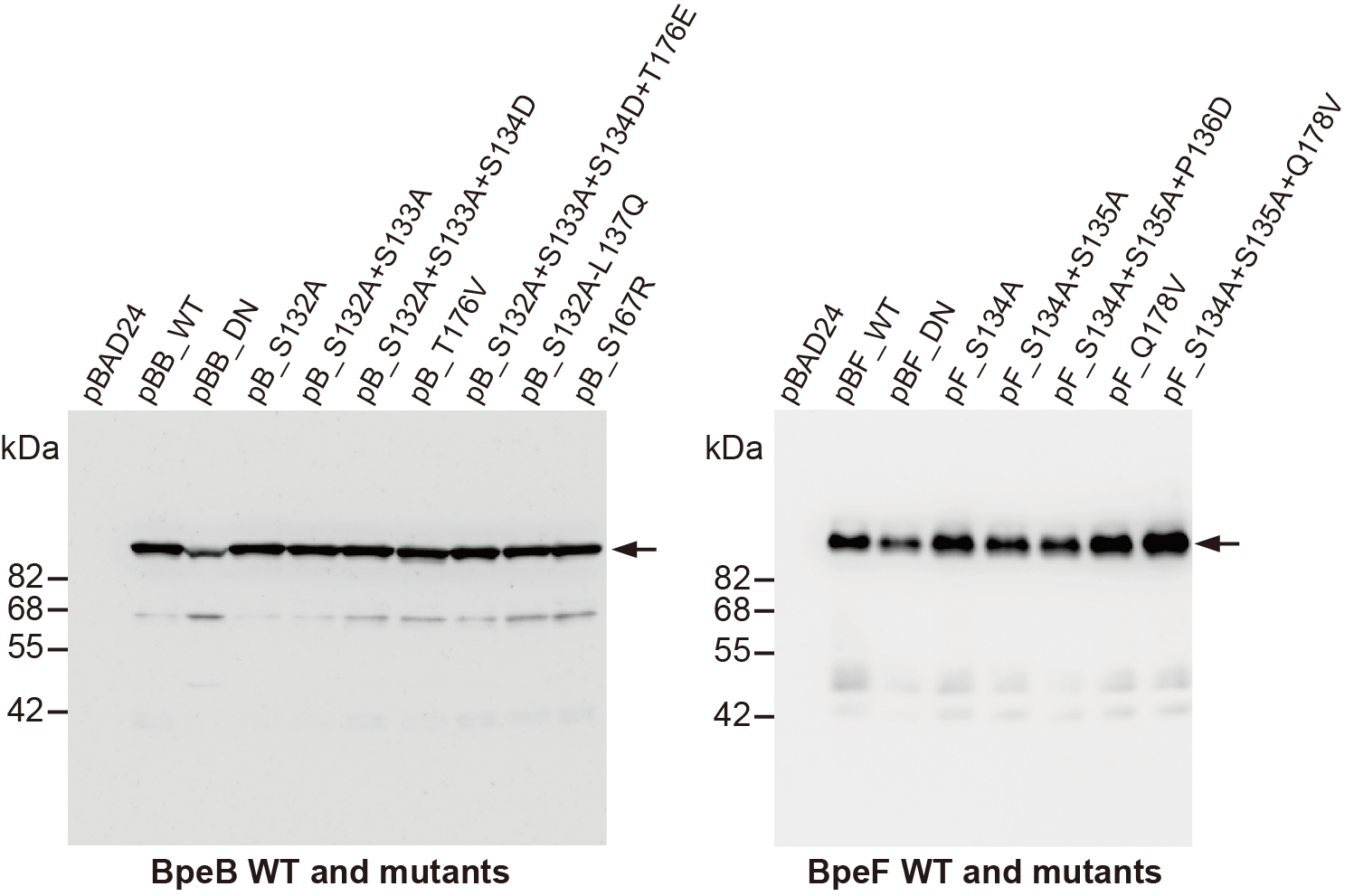


**Fig. S2 |** **Western blot analysis of the BpeB and BpeF mutants**

Aliquots of the plasma membrane fractions (10 μg of total protein) were subjected to sodium dodecyl-sulfate-polyacrylamide gel electrophoresis. The names of the plasmids contained in *E. coli* are written above each panel, and the details are provided in Table 1. Proteins were detected on immunoblots using mouse monoclonal anti-BpeB antibody or rabbit polyclonal anti-BpeF antibody as the primary antibodies and horseradish peroxidase-labeled goat anti-mouse or anti-rabbit IgG as the secondary antibodies, respectively. The arrows indicate the bands corresponding to the wild type (WT) and mutants of BpeB (left) or BpeF (right). The migration of the molecular mass markers (kDa) is indicated.


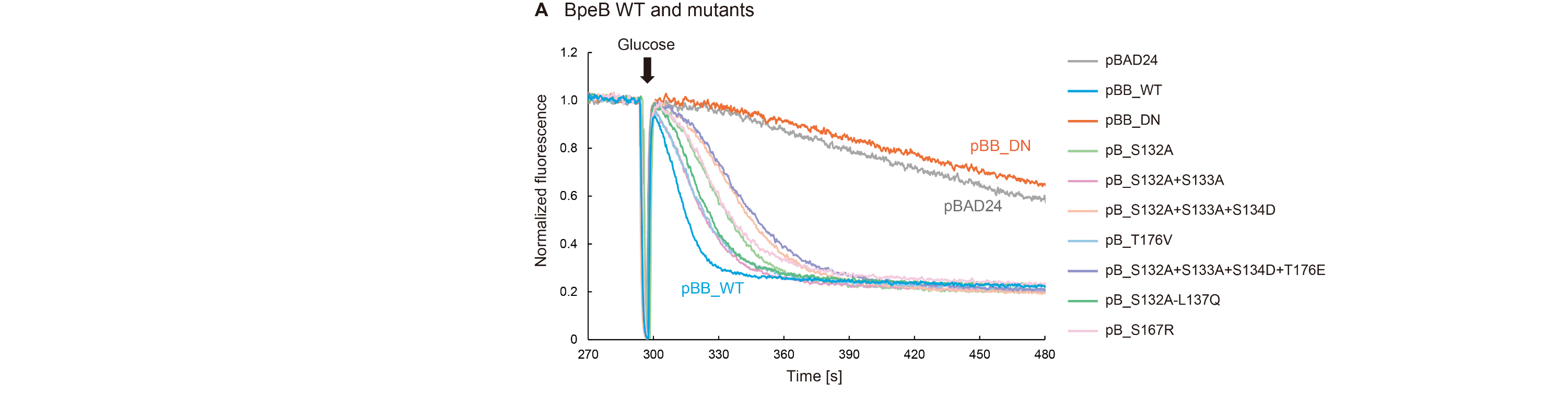


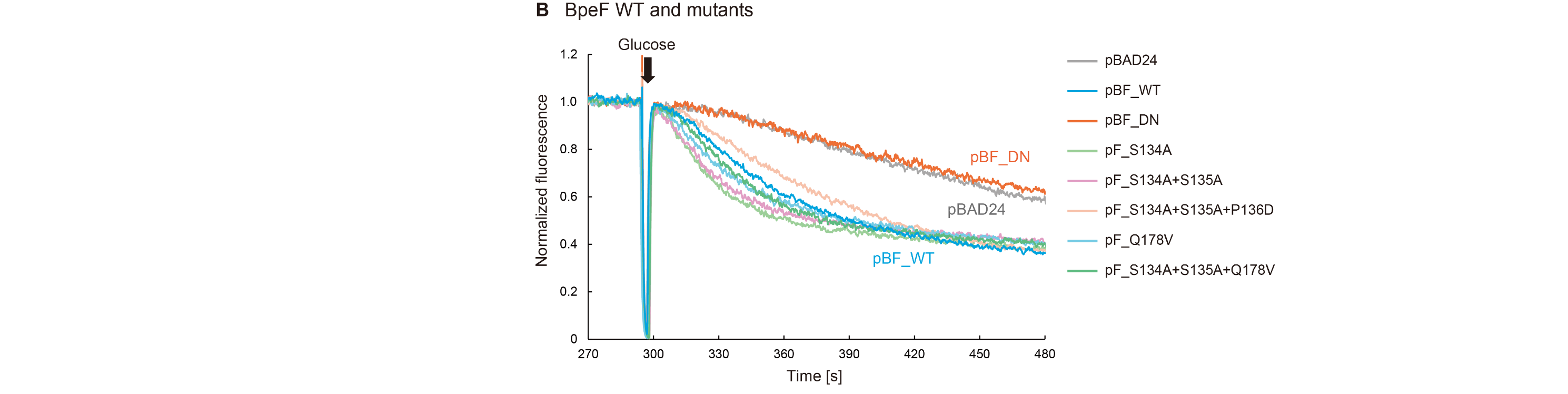


**Fig. S3 |** **Nile red efflux assay of the BpeB and BpeF mutants**

Nile red efflux was triggered at 300 s by the addition of glucose to a final concentration of 0.2% (w/v), as indicated by the black arrows. Each strain was measured at least three times.


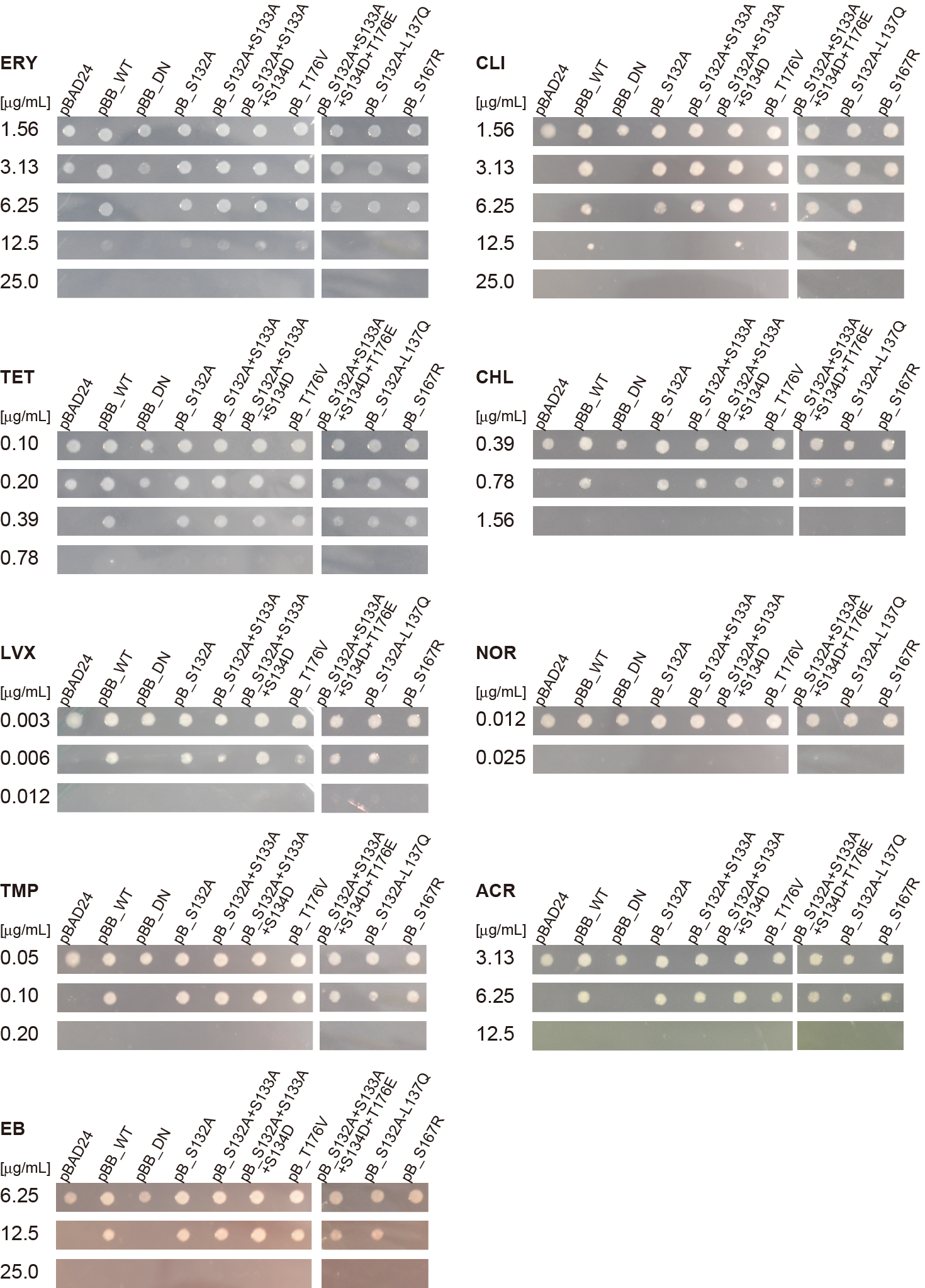


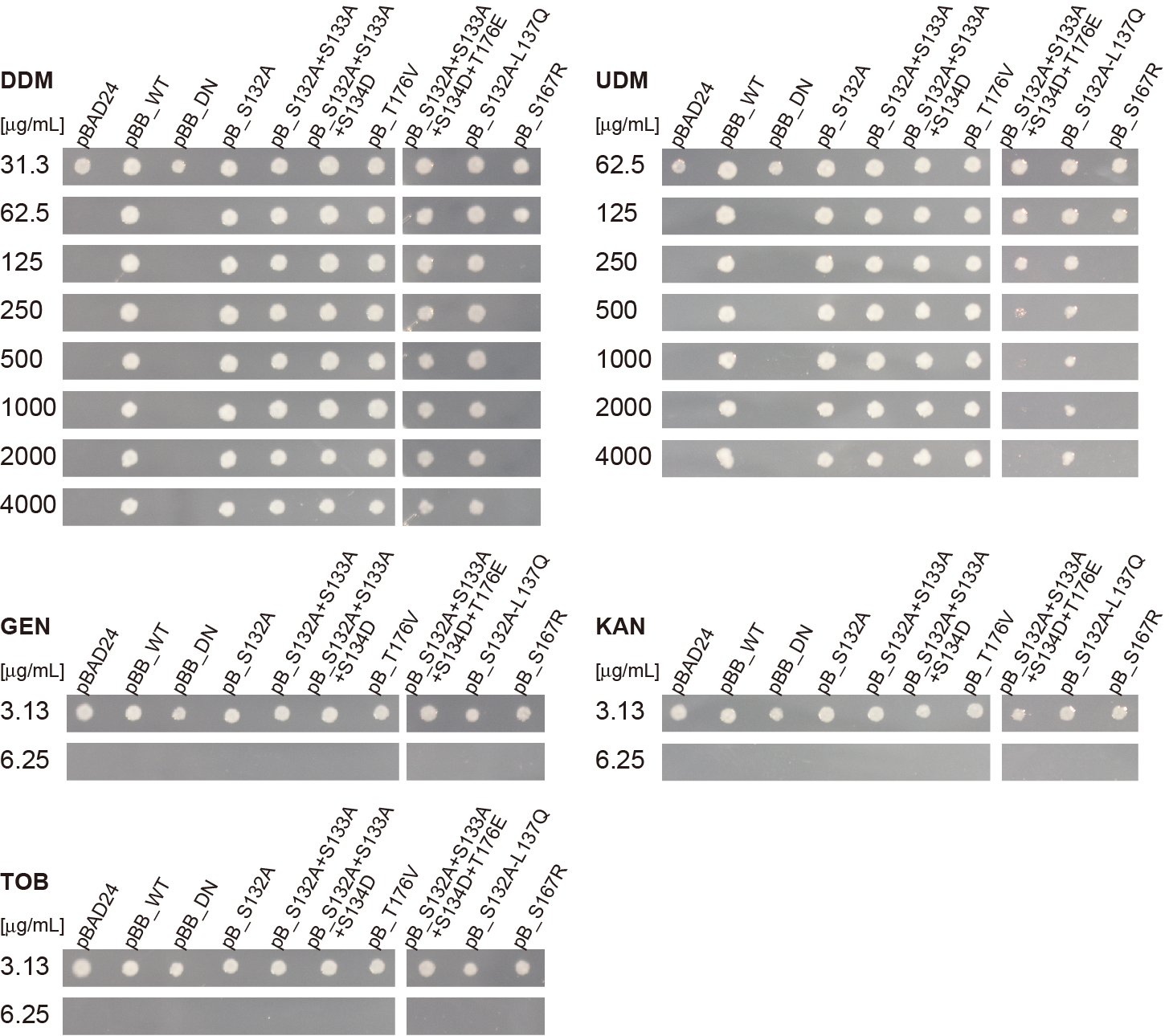


**Fig. S4 |** **Measurement of the drug resistance of *E. coli* expressing *B. pseudomallei* BpeA–BpeB–OprB and their BpeB mutants**

For the MIC measurements, cells were grown on agar plates in the presence of dilutions of the compounds. These concentrations are shown on the left-hand side of each panel. To induce protein expression, 2% (w/v) L-arabinose was added to the agar plates. The names of the plasmids contained in *E. coli* are written above each panel. Abbreviations of drugs are the same as in Figure S1, and the data in Table 2 are based on the colony formations shown in this figure.


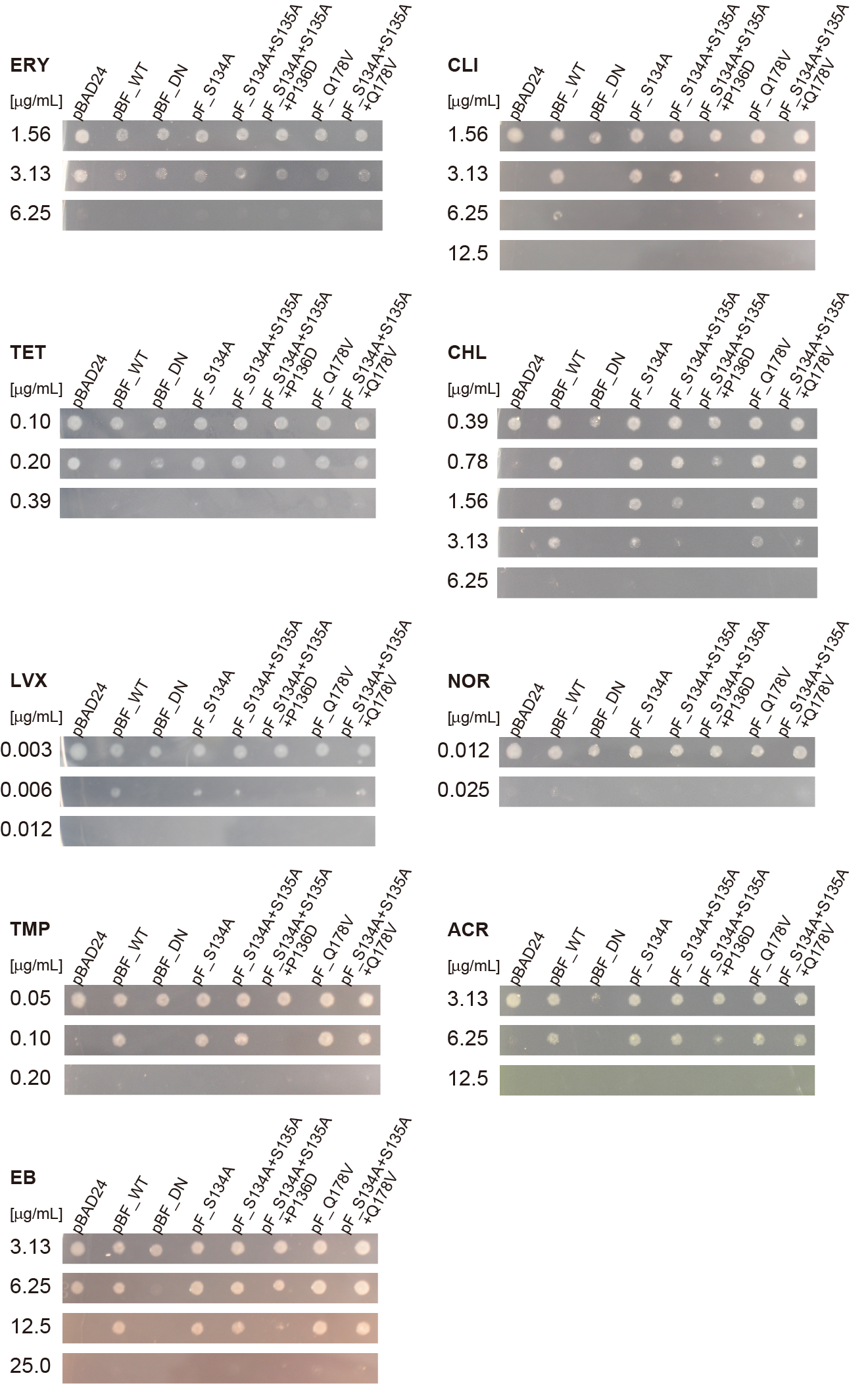


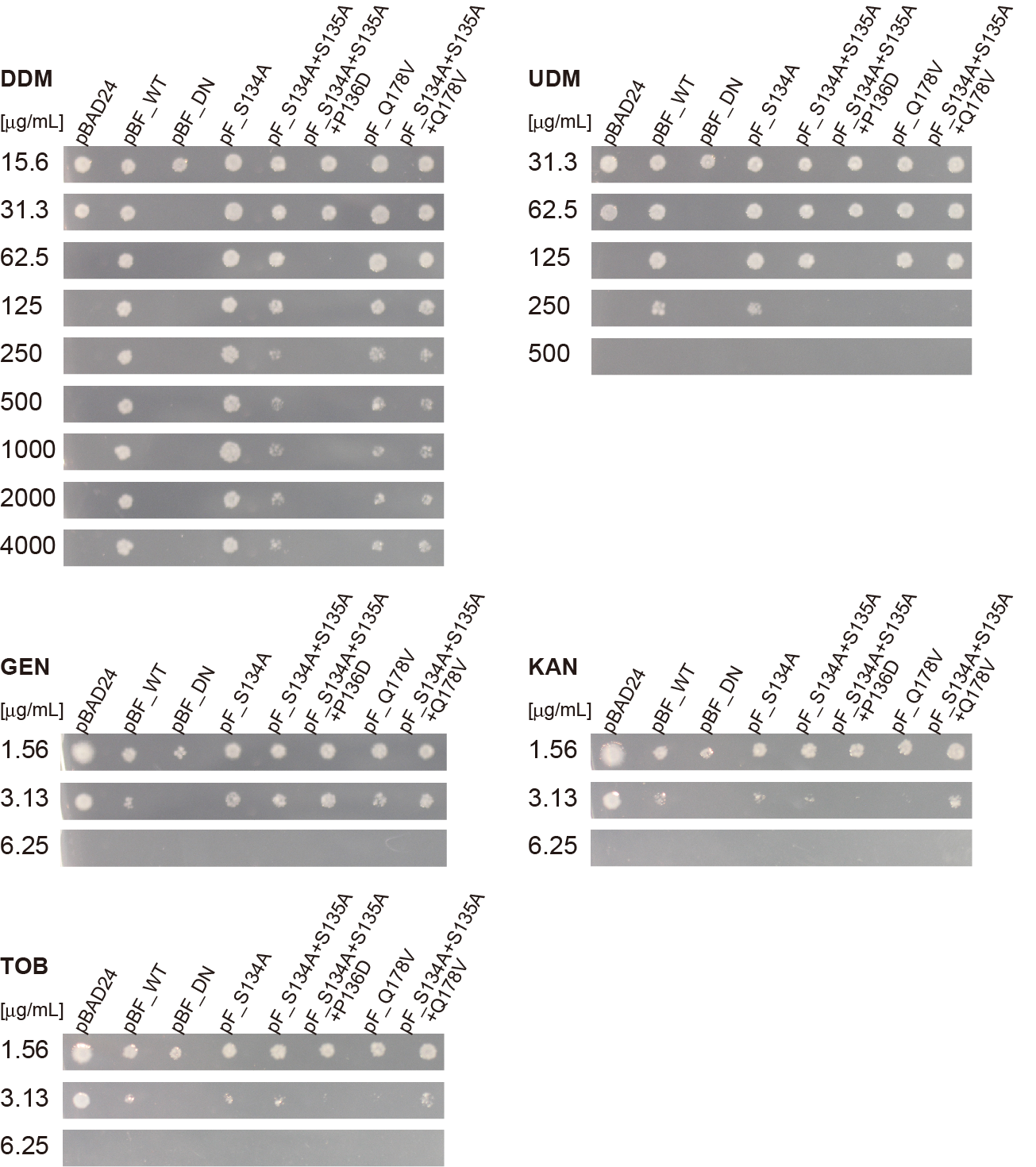


**Fig. S5 |** **Measurement of the drug resistance of *E. coli* expressing *B. pseudomallei* BpeE–BpeF–OprC and their BpeF mutants**

For the MIC measurements, cells were grown on agar plates in the presence of dilutions of the compounds. These concentrations are shown on the left-hand side of each panel. To induce protein expression, 2% (w/v) L-arabinose was added to the agar plates. The names of the plasmids contained in *E. coli* are written above each panel. Abbreviations of drugs are the same as in Figure S1, and the data in Table 2 are based on the colony formations shown in this figure.

1. **pAB_WT** and **pAB_DN (D406N and D407N)**


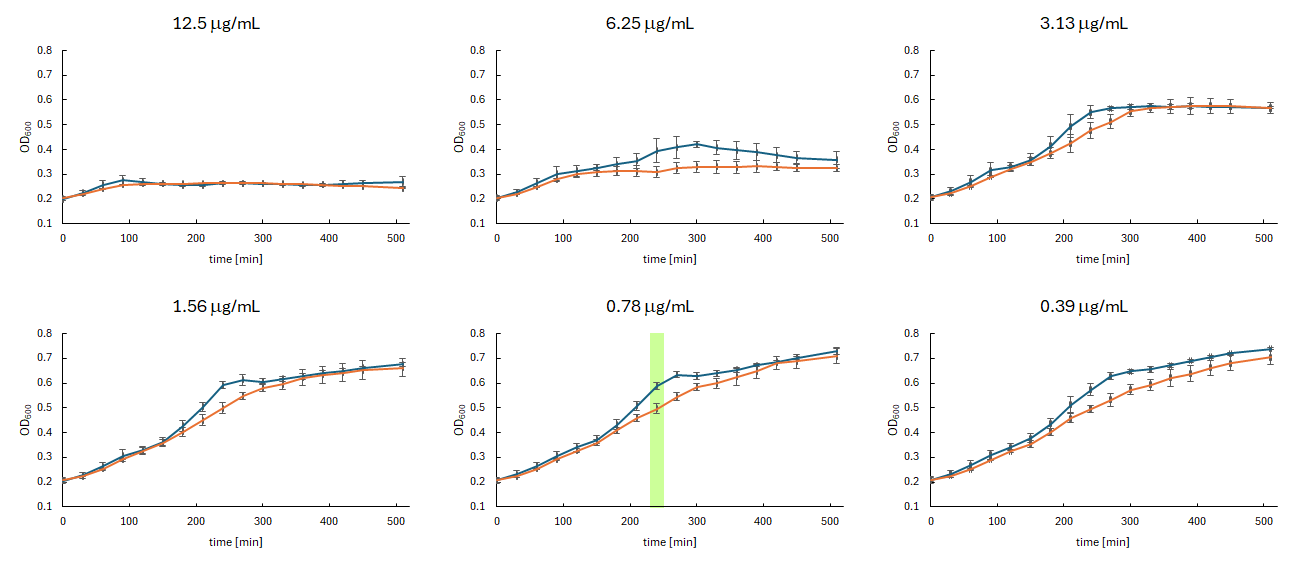


1. **pBB_WT** and **pBB_DN (D407N and D408N)**


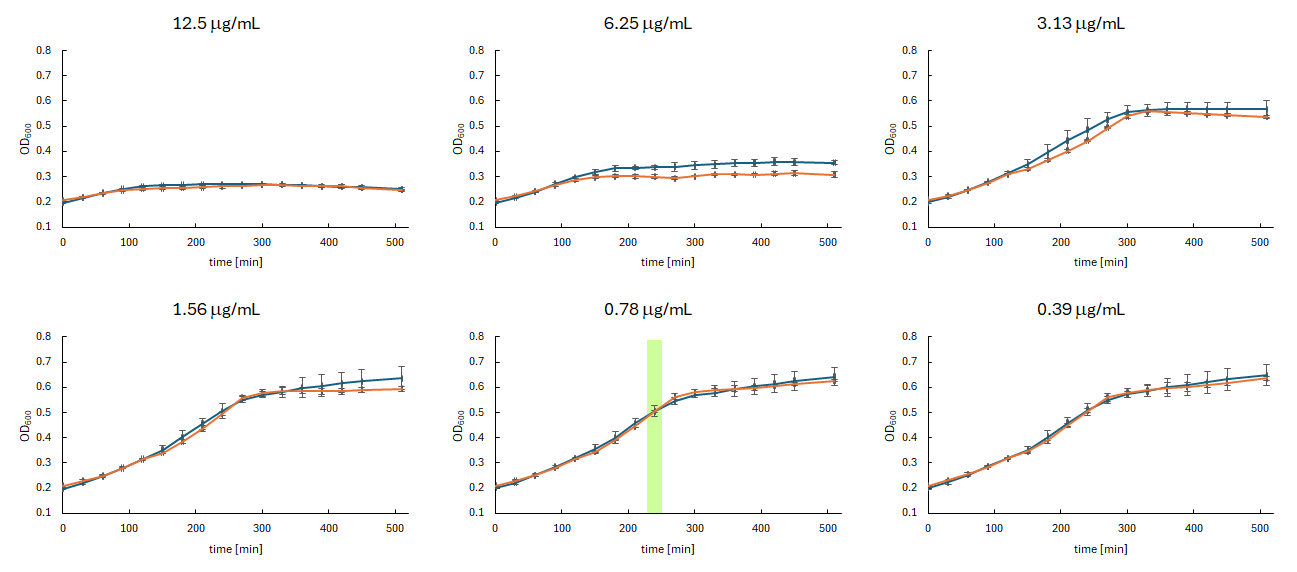


1. **pBB_WT** and **pB_S132A**


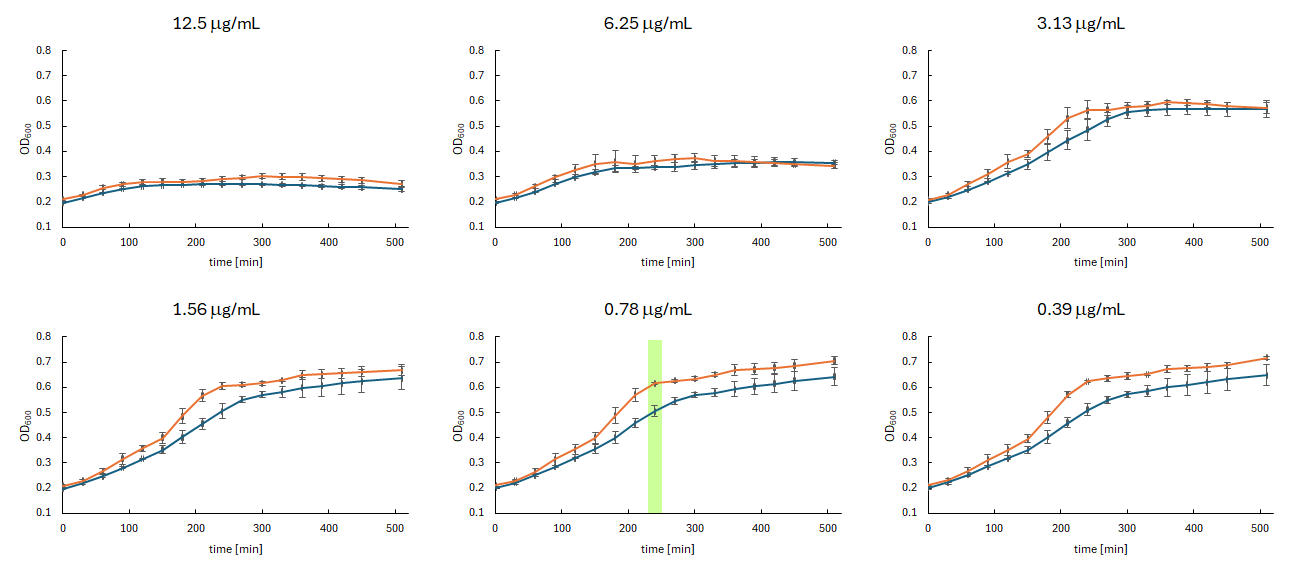


1. **pBB_WT** and **pB_S132A+S133A**


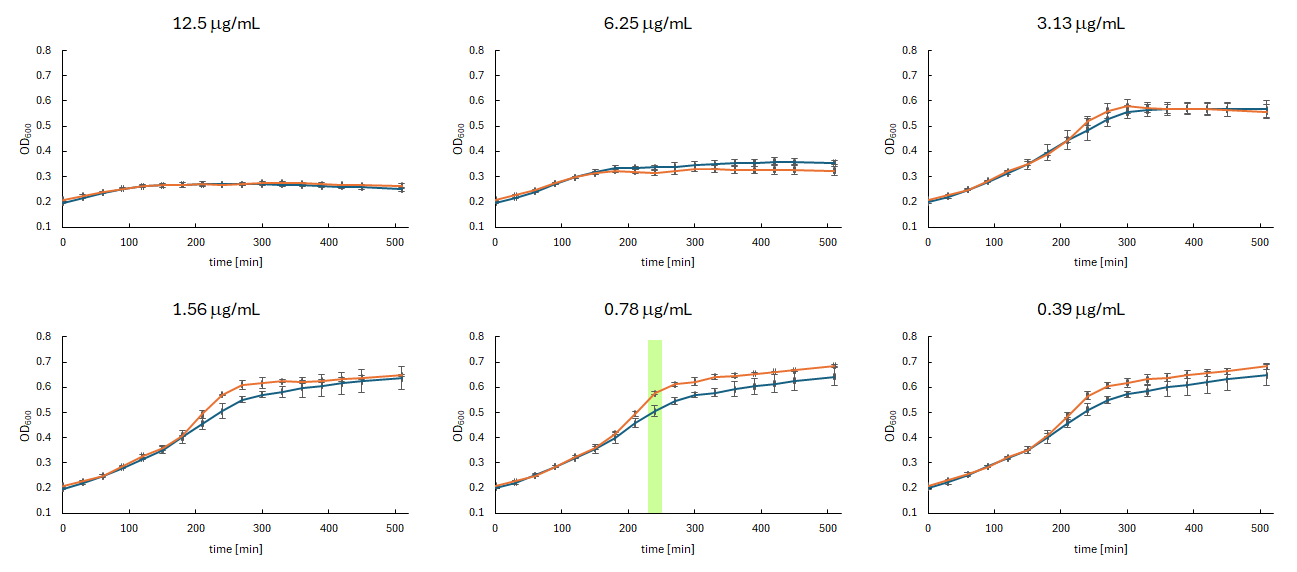


1. **pBB_WT** and **pB_S132A+S133A+S134D**


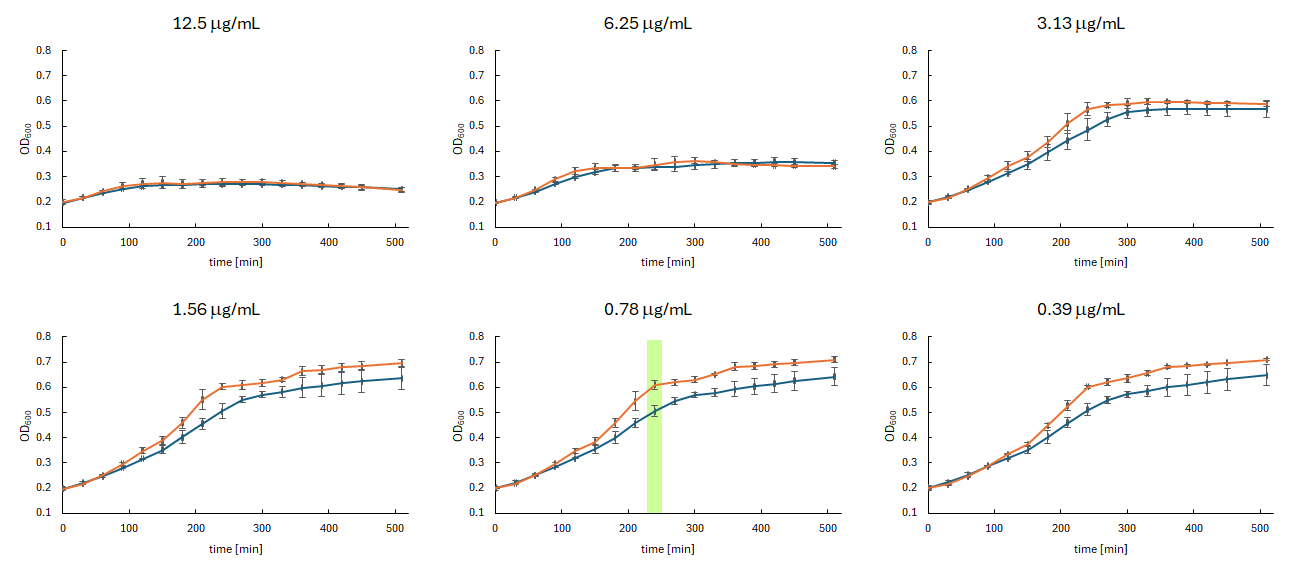


1. **pBB_WT** and **pB_T176V**


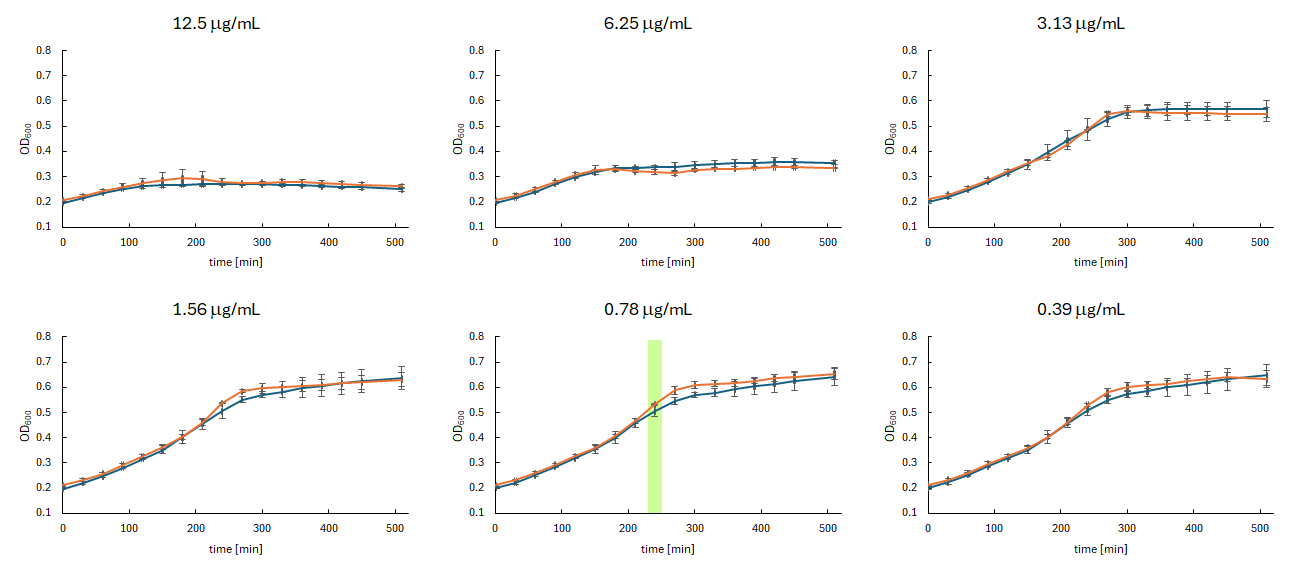


1. **pBB_WT** and **pB_S132A+S133A+S134D+T176E**


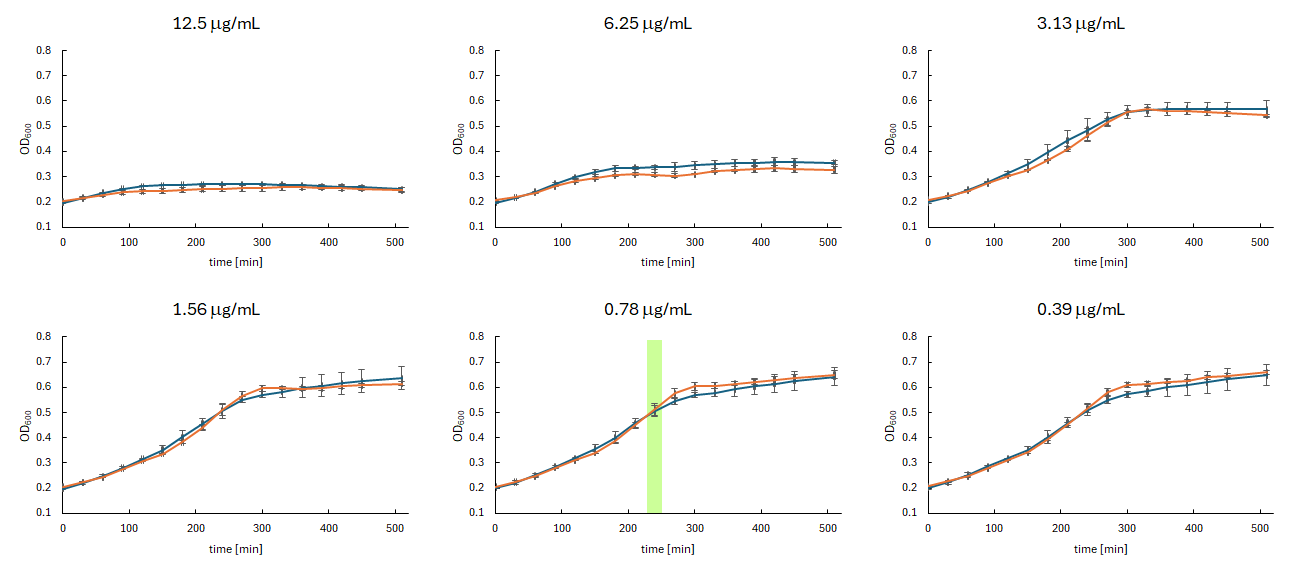


1. **pBB_WT** and **pB_S132A-L137Q (S132A, S133A, S134D, S135N, F136A, and L137Q)**


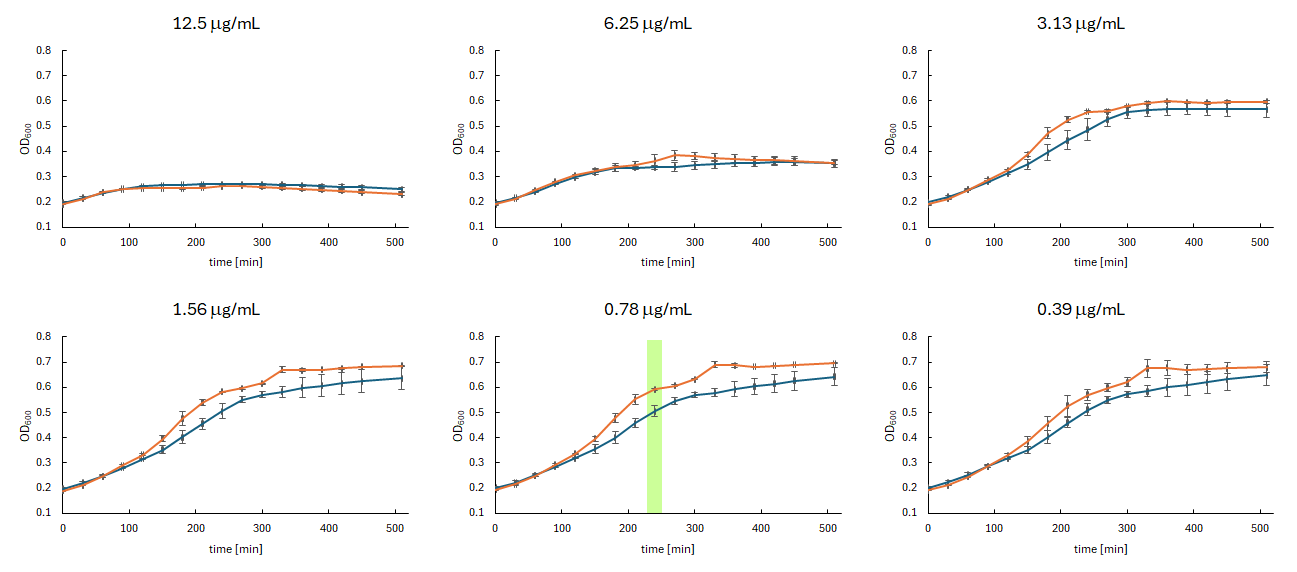


1. **pBF_WT** and **pBF_DN (D410N and D411N)**


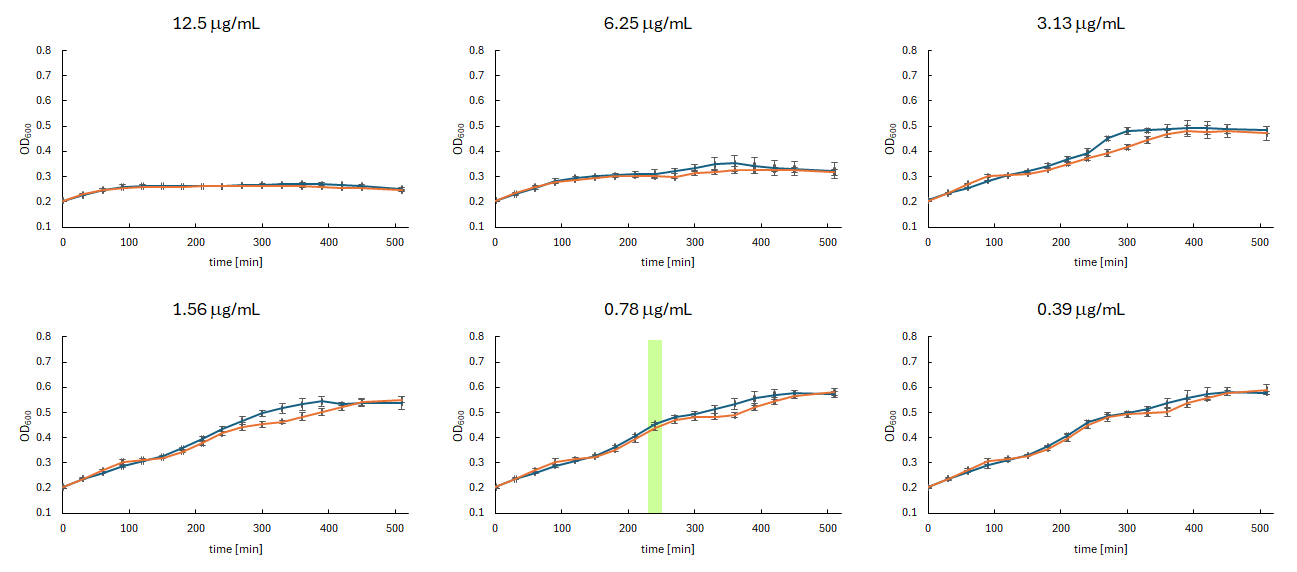


1. **pBF_WT** and **pF_S134A**


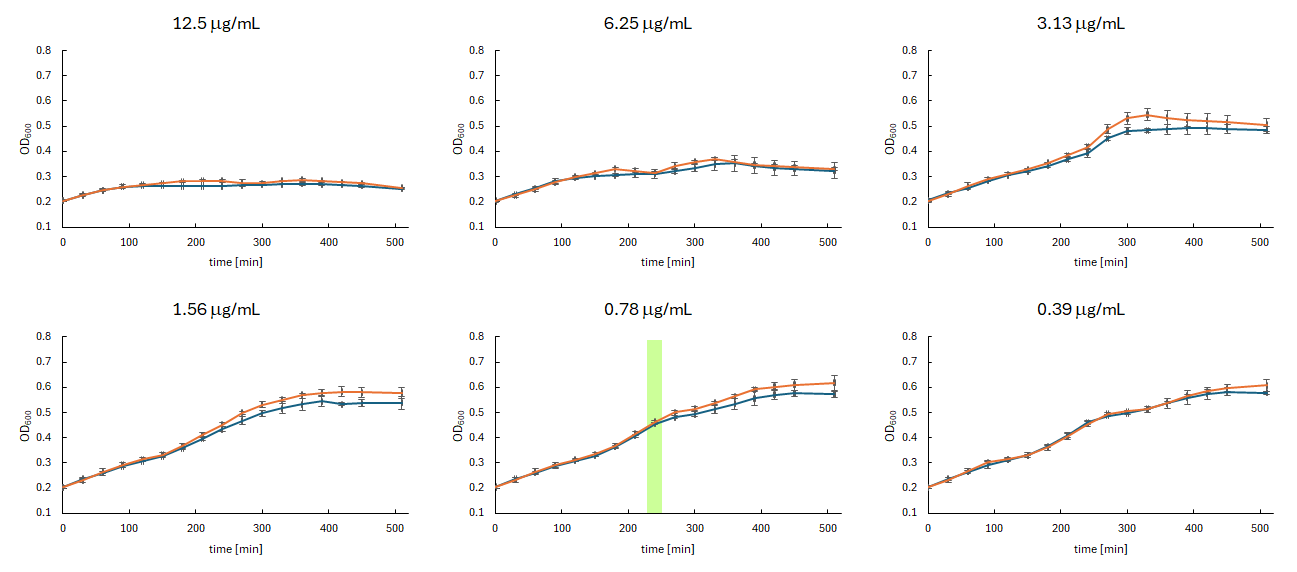


1. **pBF_WT** and **pF_S134A+S135A**


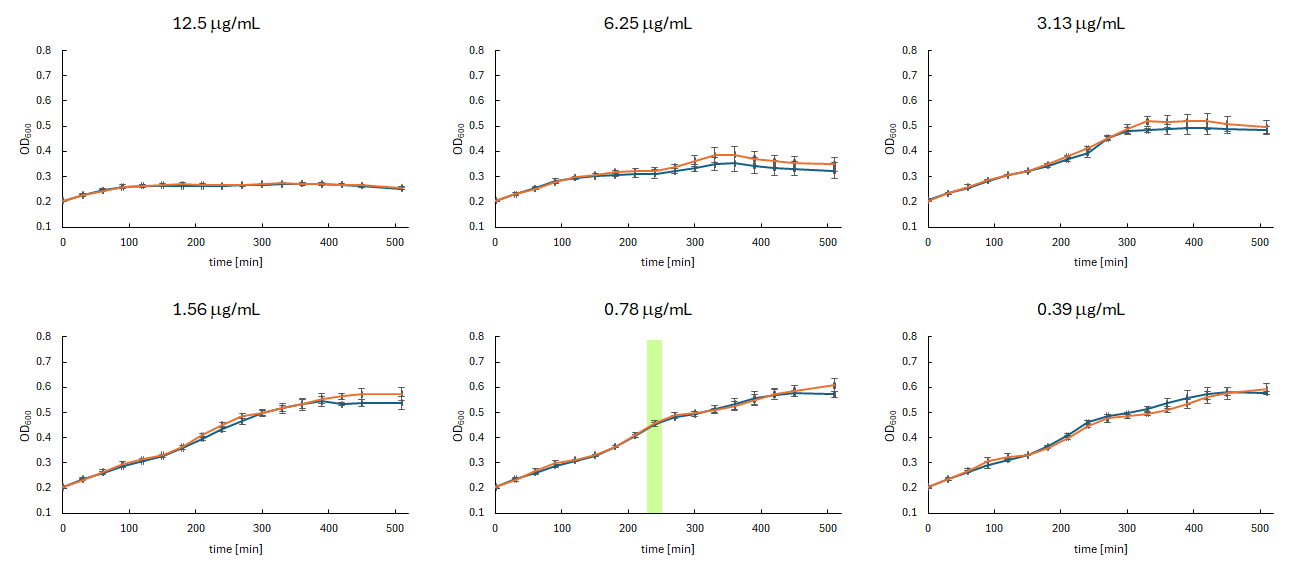


1. **pBF_WT** and **pF_Q178V**


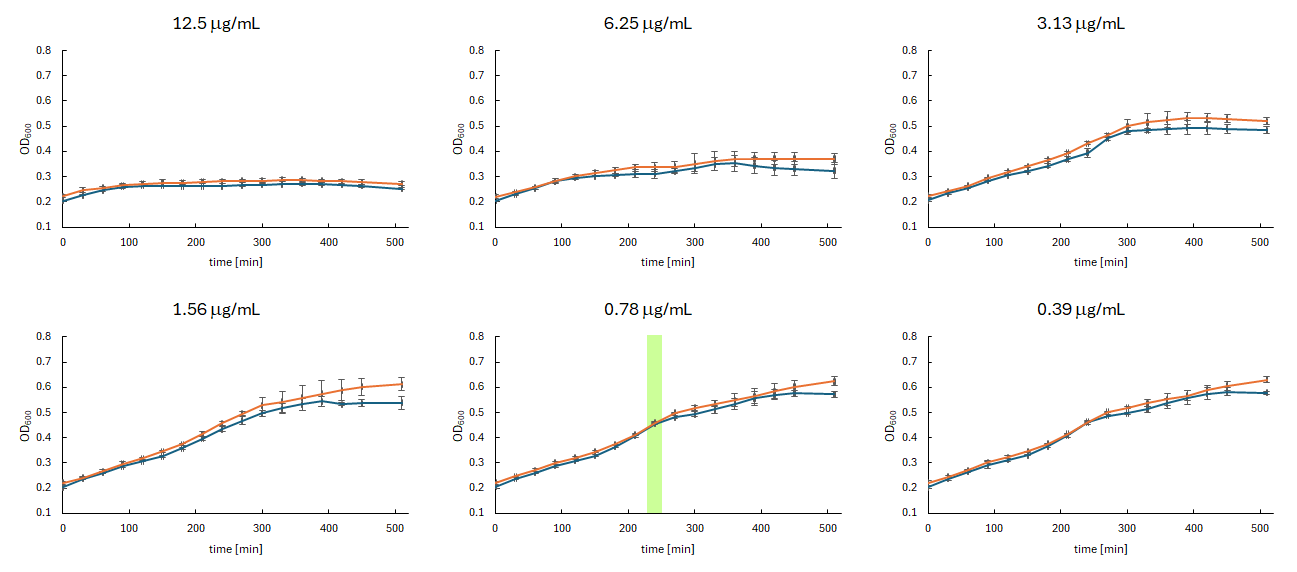


1. **pBF_WT** and **pF_S134A+S135A+Q178V**


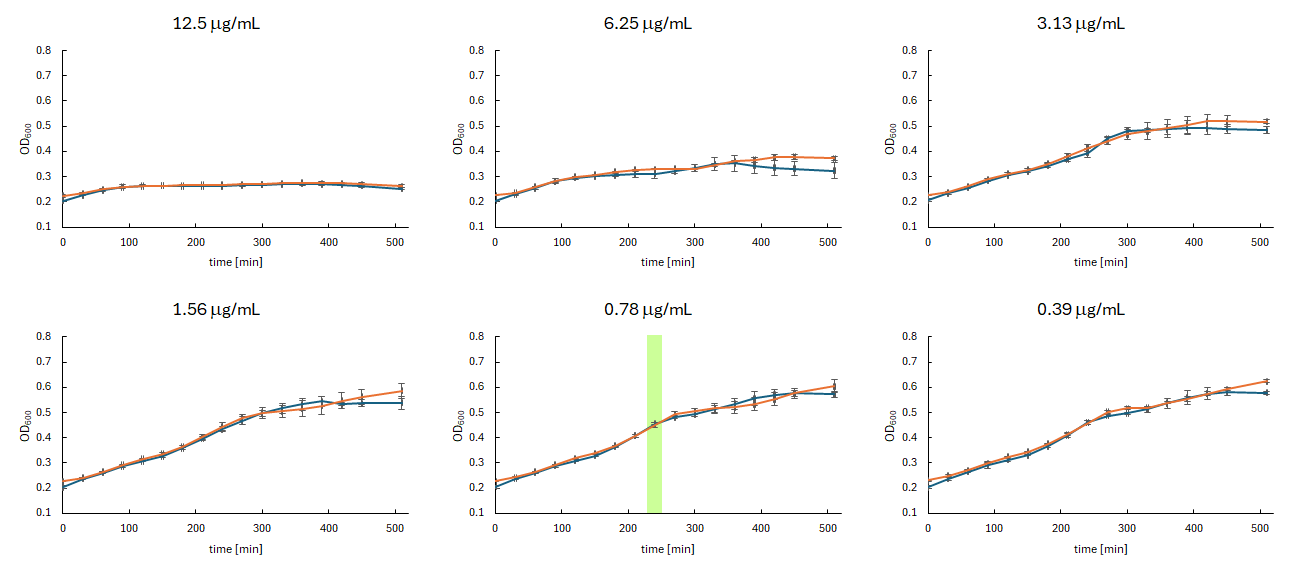


**Fig. S6 |** **Comparison of the bacterial growth curves in the presence of GEN**

The growth curves of *E. coli* W3104*ΔacrABD* harboring the plasmid expressing the WT (sky blue) and mutants (orange) in the presence of six serially diluted concentrations of GEN are shown. The names of the plasmids contained in *E. coli* are written above each panel. The light green shading indicates the data 4 h after the induction of protein expression with L-arabinose in the presence of 0.78 μg/mL of GEN used to create Figure 2. The presented data are the average of three independent experiments. Error bars indicate the standard deviation.

1. **pAB_WT** and **pAB_DN (D406N and D407N)**


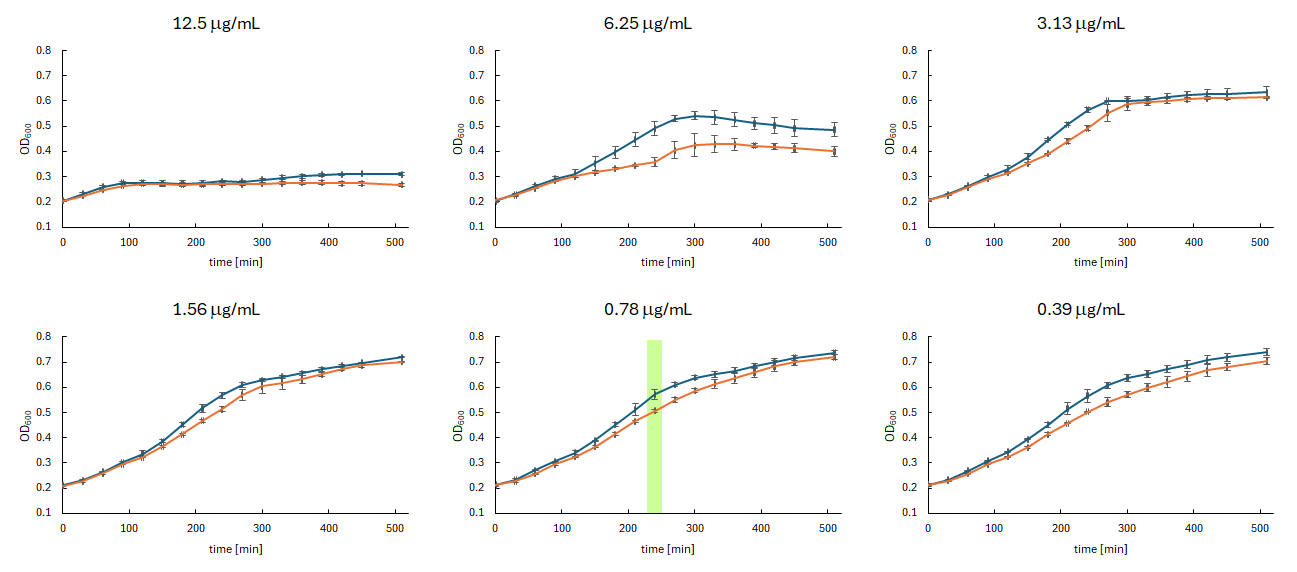


1. **pBB_WT** and **pBB_DN (D407N and D408N)**


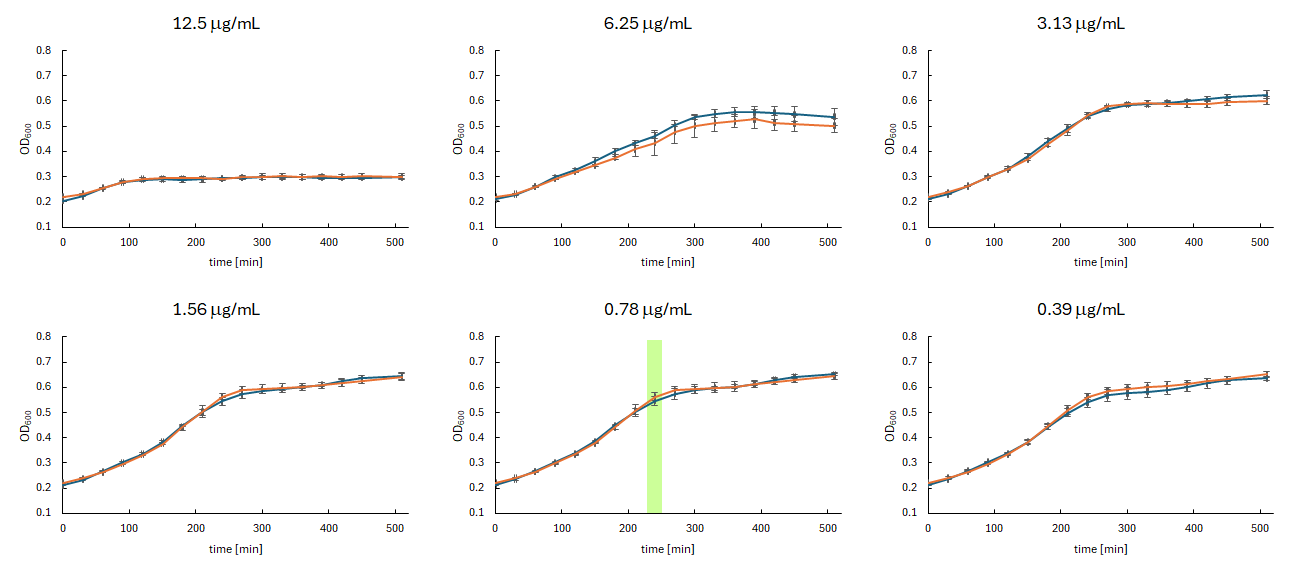


1. **pBB_WT** and **pB_S132A**


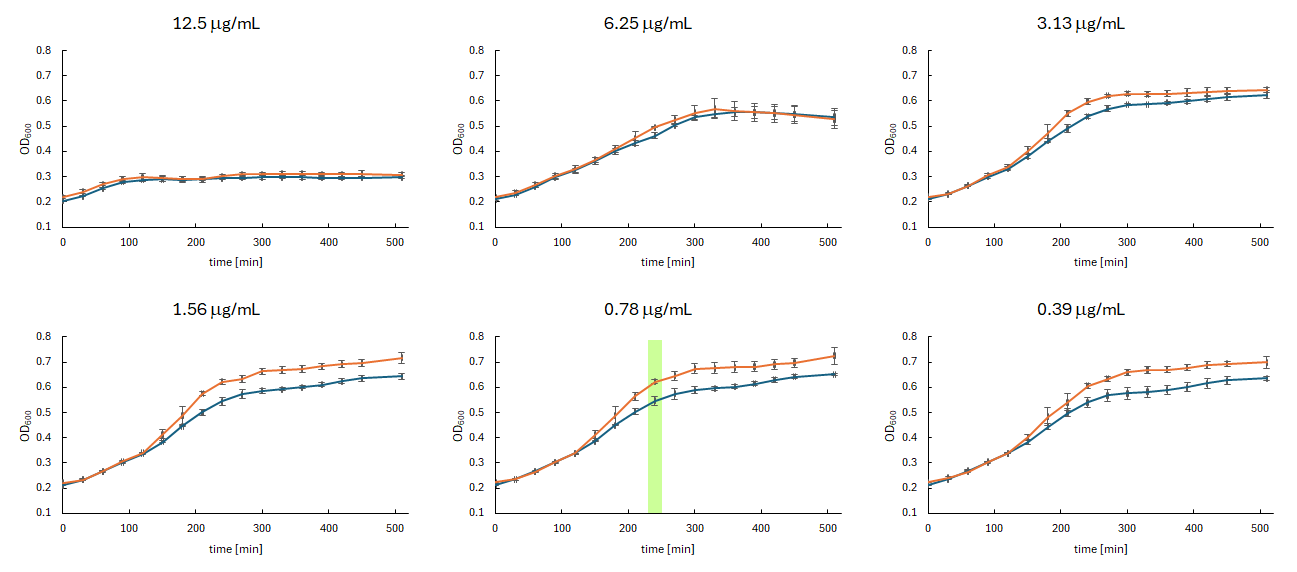


1. **pBB_WT** and **pB_S132A+S133A**


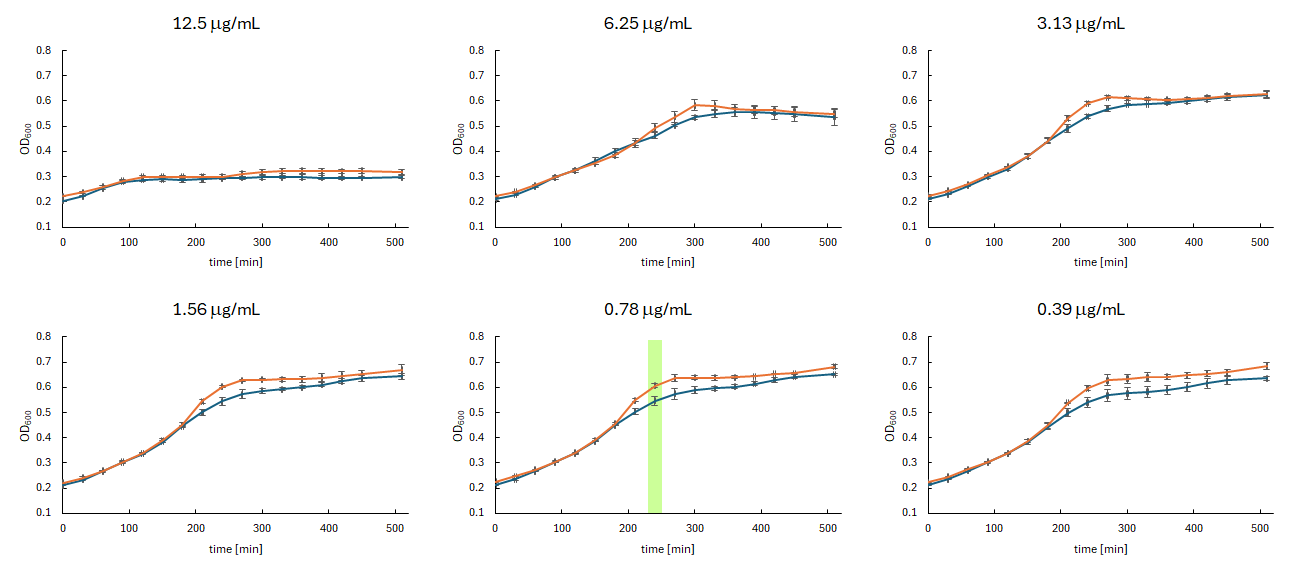


1. **pBB_WT** and **pB_S132A+S133A+S134D**


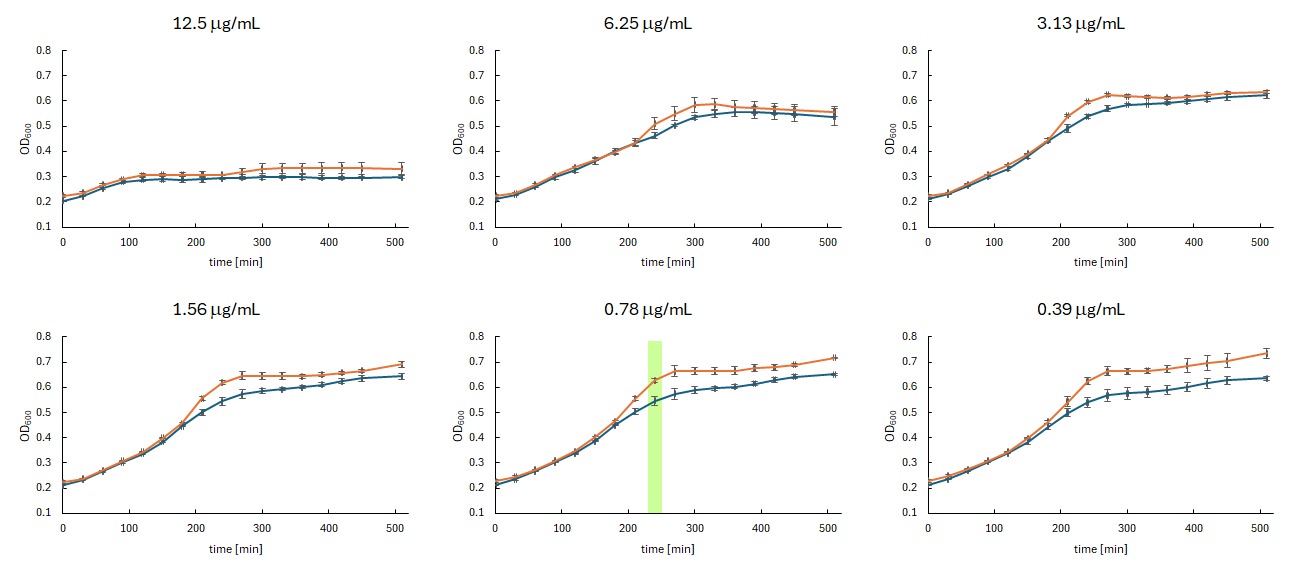


1. **pBB_WT** and **pB_T176V**


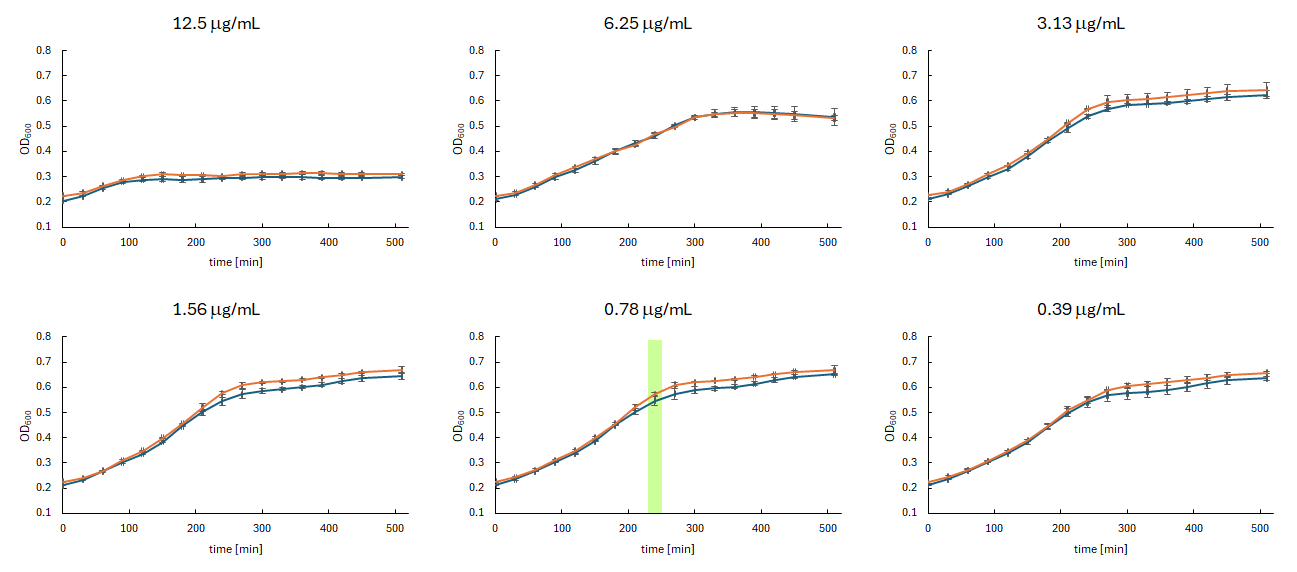


1. **pBB_WT** and **pB_S132A+S133A+S134D+T176E**


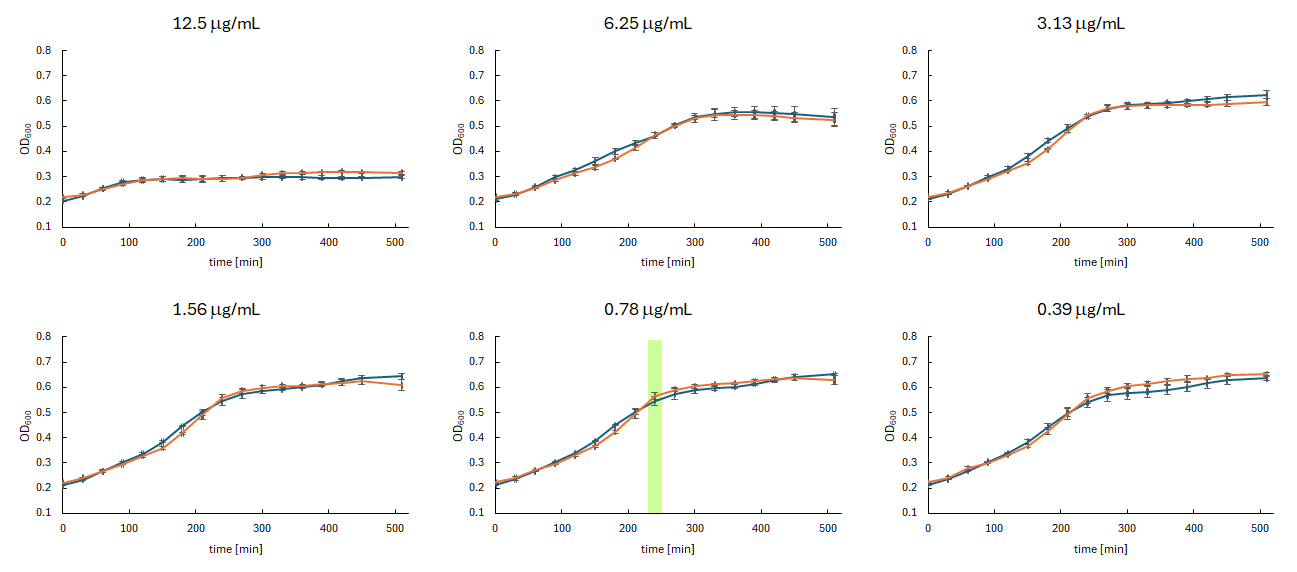


1. **pBB_WT** and **pB_S132A-L137Q (S132A, S133A, S134D, S135N, F136A, and L137Q)**


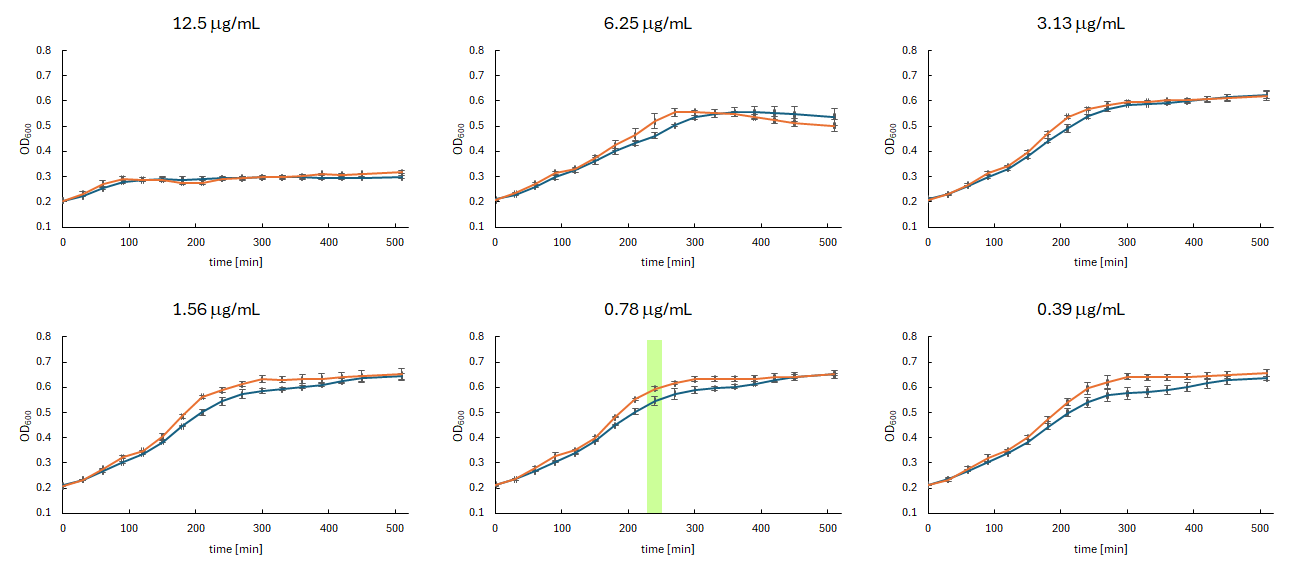


1. **pBF_WT** and **pBF_DN (D410N and D411N)**


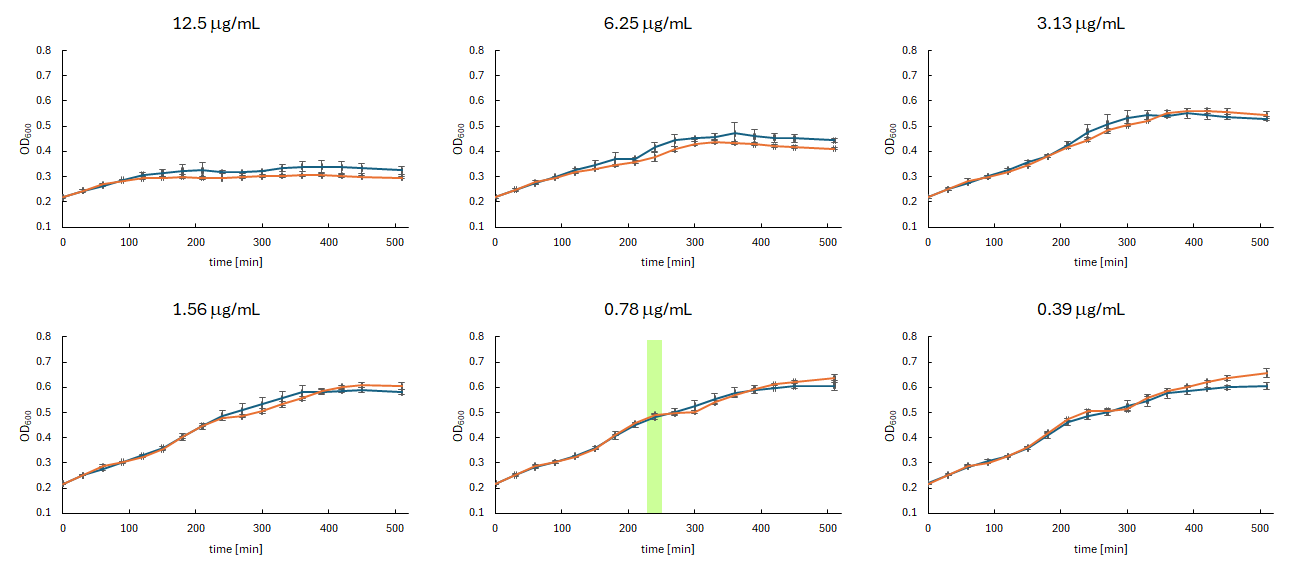


1. **pBF_WT** and **pF_S134A**


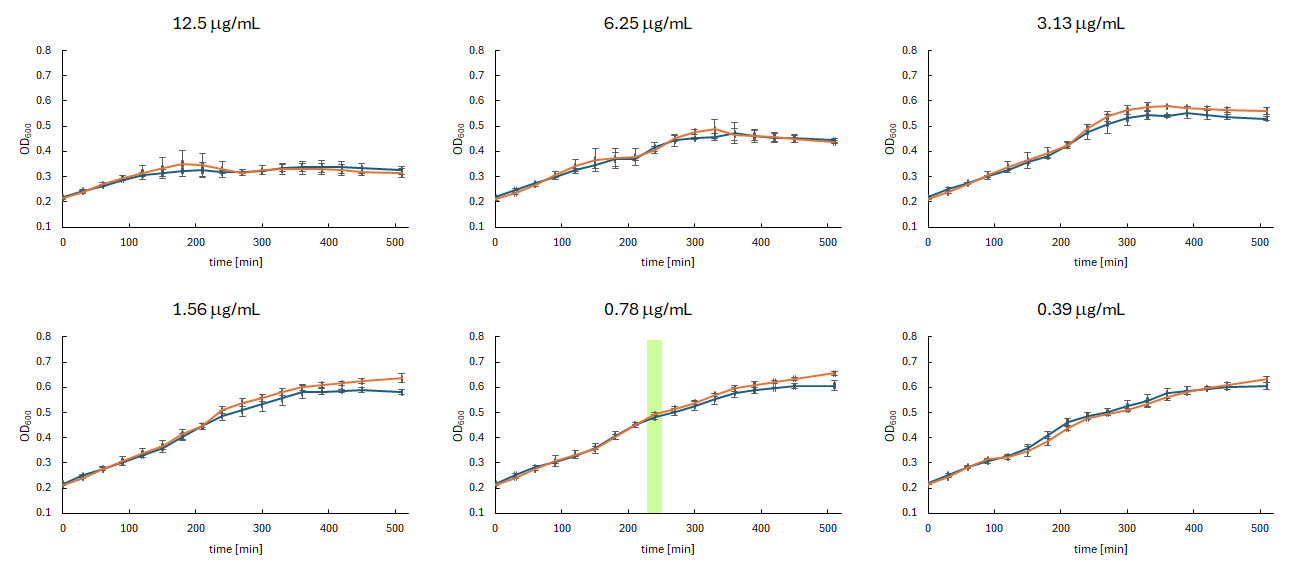


1. **pBF_WT** and **pF_S134A+S135A**


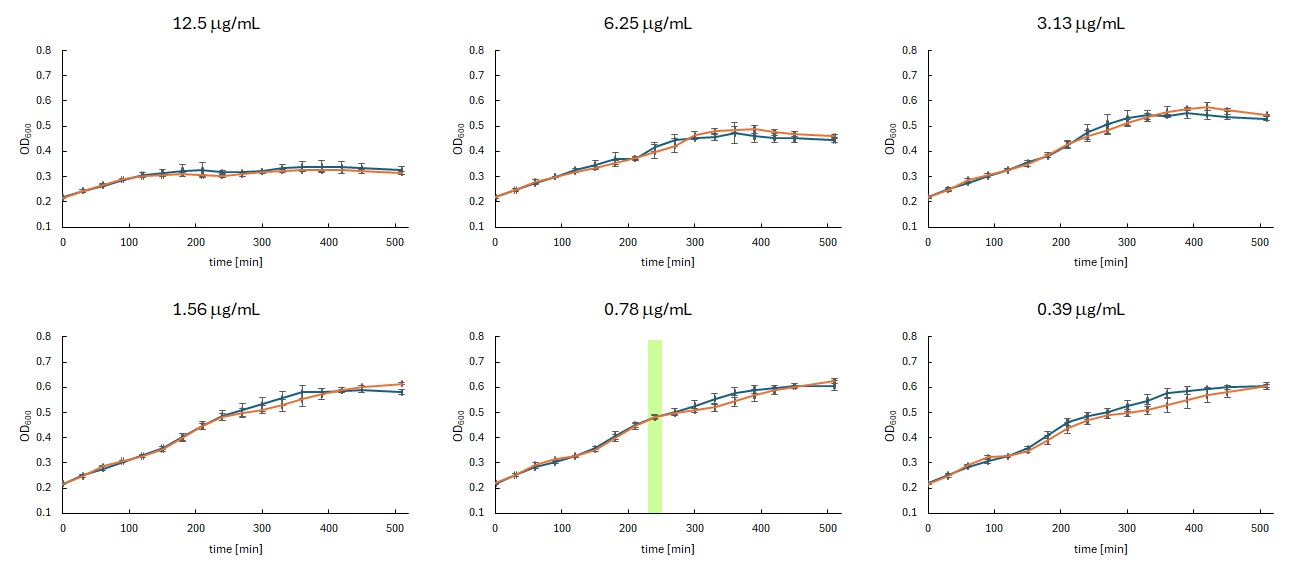


1. **pBF_WT** and **pF_Q178V**


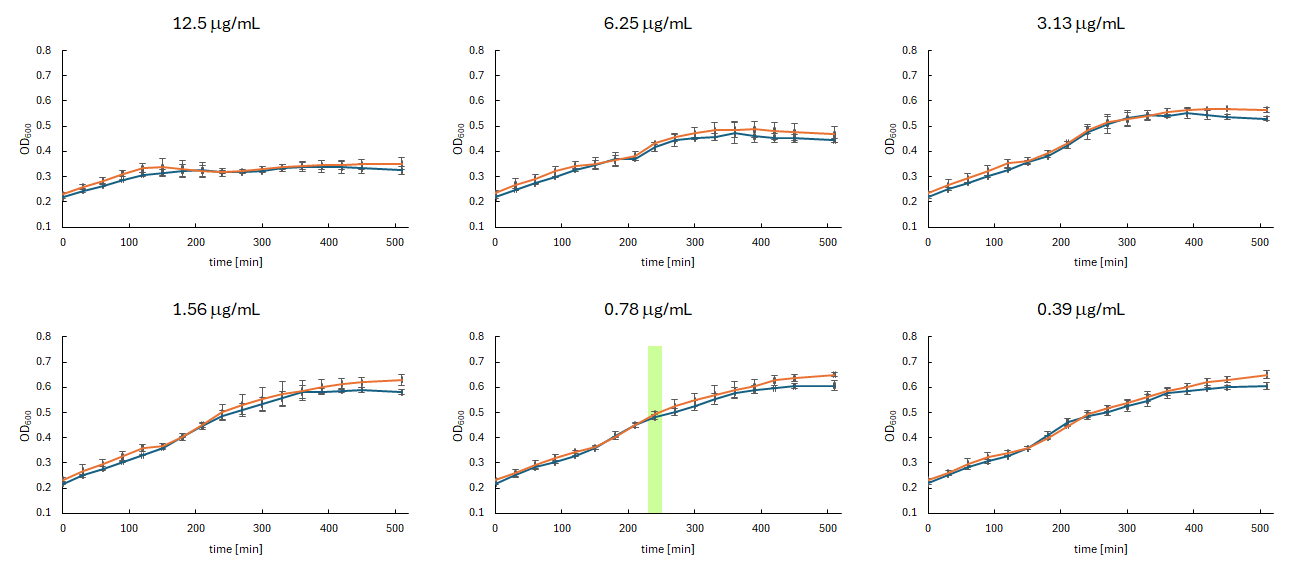


1. **pBF_WT** and **pF_S134A+S135A+Q178V**


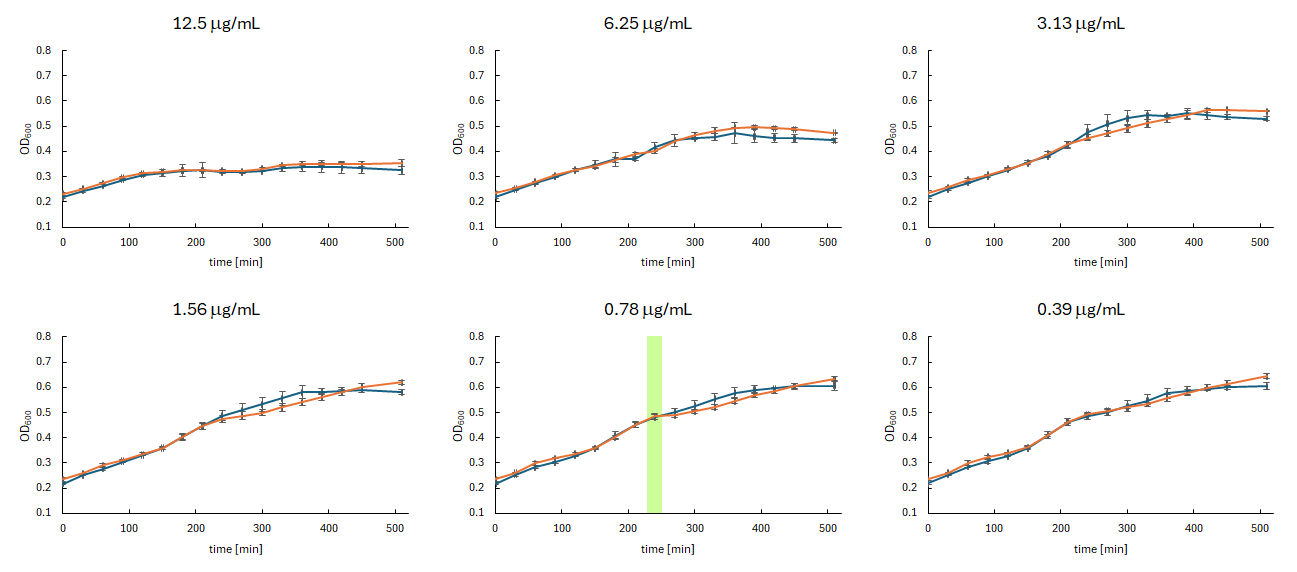


**Fig. S7 |** **Comparison of the bacterial growth curves in the presence of TOB**

The growth curves of *E. coli* W3104*ΔacrABD* harboring the plasmid expressing the WT (sky blue) and mutants (orange) in the presence of six serially diluted concentrations of TOB are shown. The names of the plasmids contained in *E. coli* are written above each panel. The light green shading indicates the data 4 h after induction of protein expression with L-arabinose in the presence of 0.78 μg/mL TOB used to create Figure 2. The presented data are the average of three independent experiments. Error bars indicate the standard deviation.

**Table S1 |** **Cloning primers**

| **Primers for gene amplification** | | |
| --- | --- | --- |
| Gene | Strand | Sequence (5′–3′) |
| *amrA* | Forward | ttgggctagcaggaggaattcaccatgaagtatgaatgggcgcgcacccg |
|  | Reverse | cagtccatggtcgggttgtgcctgtcaggttgagc |
| *amrB* | Forward | cagtccatggcccgctttttcattgaccgccccgtctttgc |
|  | Reverse | tgctctagagccgcttgcgcgccgccgcggcggcggggaacgac |
| *oprA* | Forward | tgctctagaggccaagtgaaagcgatgatgaagccccgcgcc |
|  | Reverse | ctctcatccgccaaaacagccaagcttaggcacagccatcgccaccgc |
| **Primers for site-directed mutagenesis** | | |
| Mutation in AmrB | Strand | Sequence (5′–3′) |
| D406N and D407N | Forward | gtgaataacgccattgttgtggtcgaaaatgtcg |
|  | Reverse | cagaatgccgatggccagaaccatg |
| Mutation in BpeB |  |  |
| S132A | Forward | gcgagctcgtccttcctgctggtgctgg |
|  | Reverse | tttaacggtggtgatgcccaggcgc |
| S133A | Forward | gcggcatcgtccttcctgctggtgctggcctttaac |
|  | Reverse | tttaacggtggtgatgcccaggcgc |
| S134D | Forward | gcggcagattccttcctgctggtgctggcctttaacag |
|  | Reverse | tttaacggtggtgatgcccaggcgc |
| S135N, F136A, and L137Q | Forward | caacgcgcagctggtgctggccttcaatagcgaagatg |
|  | Reverse | tctgccgccttggtgacggacaggcc |
| S167R | Forward | ccgtcgcattaatggcgtcggcaaagttg |
|  | Reverse | atcgggtccttcacgtggctggcaac |
| T176V | Forward | gttgtgctgttcggctcgcagtatgccatgc |
|  | Reverse | ggtgccgacgccattaatgcgcgag |
| T176E | Forward | gttgaactgttcggctcgcagtatgccatgc |
|  | Reverse | ggtgccgacgccattaatgcgcgag |
| Mutation in BpeF |  |  |
| S134A | Forward | gcgtcgcccaccctgaccatggttgtgc |
|  | Reverse | tttaacggtggtgatgcccaggcgc |
| S135A | Forward | gcggcgcccaccctgaccatggttgtgc |
|  | Reverse | tttaacggtggtgatgcccaggcgc |
| P136D | Forward | gcggcagataccctgaccatggttgtgcatctgatcag |
|  | Reverse | tttaacggtggtgatgcccaggcgc |
| Q178V | Forward | gtggttctgtggggcgcgggcgactatgc |
|  | Reverse | ctggccaacgccctgaatgcgcgac |

**Table S2 |** **Structural characteristics of the DBPs of RND transporters**

| Protein | PDB ID | Chain | Bound substrate | Volume  [Å^3^] | Total SASA  [Å^2^] | Polar SASA  [Å^2^] | Apolar SASA  [Å^2^] | Mean local hydrophobic density | Hydrophobicity score |
| --- | --- | --- | --- | --- | --- | --- | --- | --- | --- |
| BpeB | 7WLS | B | UDM | 1266.791 | 405.153 | 217.971 | 187.182 | 5.652 | 15.897 |
| BpeF | 7WLV | B |  | 1264.094 | 406.952 | 251.168 | 155.784 | 14.786 | 14.500 |
| AcrB | 4DX5 | B | minocycline | 958.462 | 323.797 | 162.786 | 161.010 | 15.231 | 18.750 |
| MdtF | 9QPT | C | rhodamine 6G | 926.008 | 295.140 | 184.038 | 111.102 | 1.200 | 12.231 |
| MexB | 3W9I | B | DDM | 964.042 | 331.288 | 222.602 | 108.687 | 11.200 | 16.864 |
| OqxB | 7CZ9 | A | DDM | 1184.41 | 337.080 | 234.432 | 102.648 | 10.667 | 23.051 |
| MtrD | 6VKT | B | ERY | 842.095 | 302.985 | 210.572 | 92.413 | 3.556 | 13.810 |
| MexY | 9E9F | C |  | 1032.062 | 292.768 | 144.151 | 148.617 | 32.878 | 32.227 |
| AcrD | 8F4R | A | GEN | 1154.928 | 295.918 | 228.291 | 67.627 | 5.000 | -5.722 |
| AdeB | 7KGH | C | Ethidium | 1142.042 | 292.949 | 216.868 | 76.081 | 8.889 | 2.667 |

Proteins with a hydrophilic patch in the DBP.

The volume, total solvent-accessible surface area (SASA), polar SASA, apolar SASA, mean local hydrophobic density, and hydrophobicity score of the DBPs of RND transporters with known structures were calculated using the Fpocket program [48]. Highlighted in yellow are proteins with a hydrophilic patch in the DBP. The order of the proteins is the same as in Figure 1C (excluding AmrB, for which no structure is available), and the three in the bottom row (MexY, AcrD, and AdeB) have been reported to be involved in aminoglycoside resistance. For the calculations, binding-state monomers were used after removing the coordinates of the bound substrates.
